# Climate influences the genetic structure and niche differentiation among populations of the olive field mouse *Abrothrix olivacea* (Cricetidae: Abrotrichini)

**DOI:** 10.1038/s41598-022-26937-x

**Published:** 2022-12-27

**Authors:** Marcial Quiroga-Carmona, Guillermo D’Elía

**Affiliations:** 1 Instituto de Ciencias Ambientales y Evolutivas, Facultad de Ciencias, Universidad Austral de Chile, Campus Isla Teja, Valdivia, Chile; 2 Colección de Mamíferos, Facultad de Ciencias, Universidad Austral de Chile, Campus Isla Teja, Valdivia, Chile; 3 School of Biological Sciences, University of Nebraska, Lincoln, USA

**Keywords:** Ecology, Evolution, Zoology

## Abstract

Even when environmental variation over time and space is commonly considered as an important driver of population divergence, few evaluations of intraspecific genetic variation explicitly assess whether observed structure has been caused by or is correlated with landscape heterogeneity. Several phylogeographic studies have characterized the mitochondrial diversity of *Abrothrix olivacea*, but none has incorporated landscape genetics analyses and ecological niche modeling, leaving a gap in the understanding of the species evolutionary history. Here, these aspects were addressed based on 186 single nucleotide polymorphisms, extracted from sequences of 801 bp of Cytb gene, gathered from 416 individuals collected at 103 localities in Argentina and Chile. Employing multivariate statistical analyses (gPCA, Mantel and Partial Mantel Tests, Procrustes Analysis, and RDA), associations between genetic differences and geographic and climatic distances were evaluated. Presence data was employed to estimate the potential geographic distribution of this species during historical and contemporary climatic scenarios, and to address differences among the climatic niches of their main mitochondrial lineages. The significant influence of landscape features in structuring mitochondrial variability was evidenced at different spatial scales, as well as the role of past climatic dynamics in driving geographic range shifts, mostly associated to Quaternary glaciations. Overall, these results suggest that throughout geographic range gene flow is unevenly influenced by climatic dissimilarity and the geographic distancing, and that studied lineages do not exhibit distributional signals of climatic niche conservatism. Additionally, genetic differentiation occurred by more complex evolutionary processes than mere disruption of gene flow or drift.

## Introduction

Genetic differentiation of populations is largely determined by the interplay between the homogenizing effect of gene flow and the differentiation promoted by spatially varying selection and genetic drift^1–3^. These microevolutionary processes are in turn influenced by landscape features, such as climate and topographic relief that influence dispersal patterns^4,5^. This interaction can be summarized by stating that the magnitude of the effect of landscape attributes, including their spatial and temporal dynamics, on population differentiation is strongly determined by the breadth of the fundamental ecological niche of individuals; see^6,7^. This notion is not entirely new; see^8^, but more contemporary formulations place greater emphasis on aspects of the ecological niche and as such, on selective regimes geographically structured; see^9^. For example, Rundle and Nosil^10^, and Schluter^11^, described that conspecific populations occurring in different environmental configurations may become reproductively isolated due to the adaptation of individuals to local climatic conditions. This model implies that the reproductive barrier could evolve as a pleiotropic effect of niche specialization (or niche divergence; see^12^), which would impede the existence of individuals from one population in the climatic space of the other, and hence their reproduction. Conversely, conservatism of climatic niches can prevent gene flow among populations existing in equivalent environmental configurations, when these are spatially separated by climatic conditions that are unsuitable to be crossed by individuals^13^.

Explicitly contextualizing the study of genetic structure patterns with the geographic and environmental configuration in which populations exist (i.e., landscape genetics approach, see^14^, is a useful approach for inferring the mechanisms by which these were molded; see^15,16^. However, without knowing the history of the spatial changes of population distributions, it is difficult to discern between alternative scenarios^17,18^. In such cases, the usage of ecological niche modeling (ENM) to infer the spatial changes underwent through time by a focal species or intraspecific lineage, can help to choose among alternative explanatory scenarios^19,20^. Under another approach, the contexts in which genetic differences are molded can be broadly summarized into two general models defined as isolation by distance (IBD) and isolation by environment (IBE)^21^. IBD proposes that gene flow among populations is strongly affected by the geographic distance; so, the degree of genetic differentiation between population pairs correlates with its geographic distancing^22^. In contrast, gene flow also can be impeded or reduced by the environmental dissimilarity, originating patterns that can depart from IBD^23^. Considering this fact, IBE was advanced to describe cases where genetic differentiation increases along with environmental dissimilarities, regardless of the geographic distancing populations^24^. Examination of both models should consider that geographic distance and environmental dissimilarity are often correlated, so to parsing their effects on genetic differentiation it is important to perform a parallel assessment of its association^2,21,25^.

The climate of the subtropical and temperate regions of South America (mostly those corresponding to the Southern Cone), is generally temperate with extreme spatial variation in seasonal temperature and precipitation. In arid regions such as the Atacama Desert and western Patagonia, the climatic conditions are strongly desertic, while in the coastal and foothills areas of central and southern Chile precipitations are abundant^26^. This region of South America has been strongly influenced by the interaction between Quaternary climatic variations and the Pliocene/Miocene orogenic processes^27^, which in turn has been invoked as the main driver of diversification of its current vertebrate fauna (e.g.,^28,29^). These events were also relevant for the evolution of small mammal species because its impact on demographic processes, patterns of gene flow, and changes in their geographic ranges; see^30^ for an early review. According to Pardiñas et al.^31^, the effects of these events over the structure of the mitochondrial genetic diversity of the sigmodontine rodents can be summarized in two general schemes, corresponding to a set of species that exhibit low genetic diversity and apparent absence of phylogeographic structure (e.g., *Paynomys macronyx*^32^; *Reithrodon auritus*, *Phyllotis xanthopygus*^33^, and a second set of species with high levels of genetic diversity and deep phylogeographic breaks (e.g., *Abrothrix hirta*^34^; *A*. *longipilis*^35^; *A*. *manni*^36^; *A*. *olivacea*^37^; *Loxondotomys micropus*^38^; *Oligoryzomys longicaudatus*^39^. Overall, these differences reflect the length of time that species have been resident in the area and their idiosyncratic responses to environmental events.

The olive field mouse, *Abrothrix olivacea* (Family Cricetidae, Tribe Abrotrichini), matches with the second scheme described by Pardiñas et al.^31^; its mitochondrial diversity is structured in four main and mostly allopatric phylogroups originated during the last million years, which experienced independent demographic dynamics^37^; see also^33,40–42^. However, no study has explicitly evaluated whether the identified patterns of genetic structure correlate with landscape features and their temporal dynamism. Consequently, the general implications of environmental changes on the structuring of mitochondrial diversity in *A*. *olivacea* and whether genetic differentiation within this species is associated with some landscape features remain mostly unexplored. Addressing these aspects could be considered as the next step to be taken once the pattern of genetic structure has been uncovered, given that this gap impedes to fully understand the evolutionary history of this species. *A*. *olivacea* has the broadest distribution among the sigmodontine rodents of Southern South America; see^43^, inhabiting a wide variety of ecosystems (e.g., Coastal desert, Mediterranean shrub, Valdivian and Magellanic forest, Patagonian steppe^44^), and exhibiting different levels of mitochondrial genetic structure across its geographic range^37^, making an ideal model to study the effects of the environmental variability on the genetic differentiation of small mammal species. Considering the reviewed antecedents, the aim of this study was to address the implications of dynamism and variability of the environmental-climatic features of southern South America in structuring the mitochondrial variability of *A*. *olivacea*. Past potential geographic distributions were estimated for this species and their mitochondrial lineages using ecological niche modeling, and the association between genetic differentiation and landscape features was assessed performing multivariate statistical analyses. Results obtained here suggest that gene flow among populations is unevenly influenced by the climatic dissimilarity and geographic distance, and that niche divergence observed among mitochondrial phylogroups is greater than that expected to be among intraspecific lineages. This evidence could imply that the diversification of these lineages could involve other evolutionary processes than genetic drift and/or disruption of gene flow.

## Materials and methods

### Mitochondrial data and intraspecific grouping

The analyzed mitochondrial dataset corresponds to the one assembled by Quiroga-Carmona et al.^37^ to assess the phylogeographic structure and historical demography of *Abrothrix olivacea*. The dataset consists of 801 bp of the cytochrome-b gene (*Cytb*) from a total of 416 specimens collected from 103 localities (46 from Argentina and 57 from Chile; see Supplementary materials [SM], Appendix 1). This was employed to extract a dataset of mitochondrial binary single-nucleotide polymorphisms (mt-SNP) following the workflow outlined by Jombart et al.^45^ and employed in Quiroga-Carmona et al.^37^. The mt-SNP dataset was employed to perform a genetic-based Principal Components Analysis (gPCA) to summarize the dimensionality of the mitochondrial genetic data into a reduced set of principal components axes (PCs). For this and subsequent analyses, individuals were grouped according to the four mitochondrial phylogroups (i.e., mitochondrial haplogroups) described by Quiroga-Carmona et al.^37^, which correspond to intraspecific mitochondrial lineages distributed in northern Chile (N-Ch); Mendoza, Argentina (Men-Ar); central Chile and the Chilean and Argentinian Patagonia (CS-Ch-Ar); and Tierra del Fuego Island and southernmost Chile (TdF-SCh; SM, Fig. S1).

### Genetic structure-related landscape analyses

First, the correlation between climatic dissimilarity and the geographic distance among sampled localities was explored. The 103 genetic-sampled localities were spatially projected and wrapped with a polygon created around them to represent the observed geographic distribution of *Abrothrix olivacea*. Within this polygon 5000 random spatial points were created and projected onto the 19 bioclimatic variables of the WorldClim 1.4 database^46^, employing the software QGIS, version 3.18.2-Zürich^47^. The values registered for each variable in each point were extracted with the R package *raster*^48^. This climatic dataset was employed to construct a correlation matrix based on Pearson's correlation coefficient and posteriorly analyzed with the R package *caret*^49^, to identify bioclimatic variables with more than 75% of paired correlation, to later drop them. With the geographical coordinates of each spatial point a matrix of geographic distances among populations was calculated with *raster*. Similarly, with the uncorrelated bioclimatic variables a matrix of climatic dissimilarity based on the Gower distance was calculated with the R package *vegan*^50^. These distance matrices were constructed for the whole dataset as well for each phylogroup. Matrices were employed to perform Mantel tests that were completed running 1 × 10^4^ permutations with *vegan*; these tests were completed to explore the association between the geographic distancing and the climatic dissimilarity of the sampled localities throughout the studied landscape.

The fit of the pattern of genetic structure with models of isolation by distance (IBD) and/or isolation by environment (IBE) was initially evaluated using matrices of genetic, geographic, and environmental distances calculated between pairs of localities. The genetic distances were calculated as *p-*distance for the *Cytb* sequences using MEGA X 10.1.8^51^, while the geographic and climatic distances were those specified above. Distance matrices were employed to perform Mantel and Partial Mantel tests employing *vegan* with 1 × 10^4^ permutations. The matrix of genetic distances was correlated with the matrices of geographic distances and climatic distances. These analyses were developed both for the total dataset corresponding to *Abrothrix olivacea* and for the datasets corresponding to each mitochondrial phylogroup; except the Men-Ar phylogroup due to it includes only two localities.

Differences among the magnitude of the IBD observed for each mitochondrial phylogroup were explored using a Procrustes analysis (PA^52,53^). This analysis consisted in the superimposition of a genetic map constructed with the projection of 416 individuals onto the orthogonal intersection of the two first principal components of the gPCA, onto a geographic map where the 103 collection localities of the individuals are projected. Geographical coordinates were converted onto a conical projection with the R package *rgdal*^54^, and the genetic and geographical maps were superimposed with *vegan*. The significance of the Procrustes similarity statistic (*t*_*0*_) was assessed with 1 × 10^4^ randomized permutations of locality placement, computing the similarities scores (*t*) between the gPCA coordinates and the geographic locations. Then, the probability of observing a similarity statistic higher than *t*_*0*_ under the null hypothesis (i.e., no geographic pattern exists in the population structure) was calculated; see^53^. Finally, an analysis of variance (ANOVA) was performed using the magnitude of the residuals to test whether deviations from the expected pattern of genetic variation based on geography are different among the mitochondrial phylogroups.

Finally, to identify the climatic features that are associated with the structure of the mitochondrial variability of *Abrothrix olivacea* a Partial Redundancy Analysis (RDA) was conducted. This ordination method is used in landscape genetics to evaluate the IBE model because it minimizes type I errors when used to inspect landscape genetic relationships^55^. This is a constrained ordination method comparable to a linear regression that can be used to model multivariate response data and distinguish the relative contribution of landscape features (e.g., as climatic aspects) and the spatial components on genetic structure; see^56^. The coordinates of the individuals in the first 20 PC of the gPCA were employed as genetic-response variables, and the previously identified uncorrelated bioclimatic variables were employed as landscape-predictor variables. In addition, the geographic coordinates (latitude and longitude) of each sampling locality were included as conditional variables to consider the effect of the geographic distancing on the pattern of genetic structure. The RDA was completed with a stepwise model selection to identify the climatic predictor variables that best explain the genetic differences among individuals. Subsequently, ANOVAs were performed to assess the significance of constraints and the RDA axes. These analyses were completed with *vegan*.

### Climatic characterizations and niche differentiation

The differentiation of the climatic niche of the mitochondrial phylogroups of *Abrothrix olivacea* was addressed characterizing the climatic attributes of their geographic ranges. In this case, the localities corresponding to each phylogroup (N-Ch = 14, CS-Ch-Ar = 73, TdF-SCh = 17) were used to extract the values registered for each bioclimatic variable (employing steps described in the previous section); except for the phylogroup from Mendoza, Argentina, as it was sampled only at two localities (see SM, Fig. S1). These climatic datasets were pooled and analyzed implementing a Principal Component Analysis (PCA) performed with a correlation matrix and using the R package *FactoMineR*^57^. The significance of climatic differences between phylogroup pairs was assessed with a Pairwise Permutational Multivariate Analysis of Variance (PERMANOVA) of Euclidean distances with 1 × 10^4^ iterations, using *vegan*. Posteriorly, locality scores in the first three PC were used to construct Gaussian hypervolumes to represent the climatic niche of each mitochondrial phylogroup, and theses ellipsoid were them projected in a common multidimensional climatic space to evaluate its degree of differentiation; see^58^. This procedure was completed employing the R package *hypervolume*^59^, which was also used to compute the Jaccard and Sørensen indexes as measures of niche comparison and whose values of similarity range from 0 (fully different) to 1 (fully similar).

### Ecological niche modeling and reconstruction of past distributions

The ecological niche models (hereafter referred as niche models) were developed to evaluate the importance of the climatic dynamism of the Quaternary on the differentiation of the mitochondrial genetic diversity of *Abrothrix olivacea*. The past geographic distributions of this species and their mitochondrial phylogroups were reconstructed by estimating the geographic distribution of the climatically suitable areas available during the Last Interglacial (LIG: ∼120–140 Kay BP), the Last Glacial Maximum (LGM: ∼22 Kay BP), and the Mid Holocene (Mid-Holo: ∼6–8 Kay BP). The climatic niches were modeled using as primary presence data the 103 localities where the sequenced individuals were captured. This occurrence dataset was enriched using the records available at the Global Biodiversity Information Facility (GBIF; http://www.gbif.org/), which strictly came from specimens deposited in biological collections. This second dataset was spatially filtered with the R package *spThin*^60^ and using a threshold of 15 km of separation between localities to minimize the potential effects of spatial autocorrelation^60^. The filtered records were pooled with the 103 initial localities, and subsequently these records were partitioned according to the geographic distribution of each mitochondrial phylogroups (SM, Fig. S2). Posteriorly, employing *raster* a study region was established for each modeling instance (i.e., the climatic niche of *A*. *olivacea* and that of its mitochondrial phylogroups), delimiting areas adjusted to the minimum convex polygon formed by the corresponding presence records, plus the addition of an enveloping geographic buffer of 50 km (SM, Fig. S2). The bioclimatic variables obtained from WorldClim 1.4. were employed as environmental predictors, but only were included in the niche modeling process those that were not correlated with each other (as identified in the previous section); a necessary precaution when projecting niche models into different spatial and/or temporal scenarios (i.e., model transference^61^). Thus, the following six variables were employed for modeling climatic niches: Bio 03: isothermality ([Bio 2/Bio 7] × 100), Bio 04: temperature seasonality, Bio 06: min temperature of coldest month, Bio 09: mean temperature of driest quarter, Bio 15: precipitation seasonality, and Bio 16: precipitation of wettest quarter. These variables are good descriptors of the extreme climatic conditions and their strong seasonal fluctuations throughout the study region (Chile and Argentina; see^62^) and at the same time represent adequate surrogates for climatic factors limiting the dispersion and distribution of terrestrial mammal species, see^63,64^.

The niche models were constructed using the maximum entropy method as implemented in MaxEnt, version 3.4.4^65–67^. Optimal model performance and complexity were estimated for each set of presence data, varying the values of regularization multiplier (rm) from 0.5 to 4, with increments of 0.5, and the feature classes (fc) used in each modeling instance. These procedures were completed employing the *ENMeval* R package^68^ and selecting a spatial block approach as the scheme for the geographic partition, see^48,69^. The final setting for each model was selected based on the values of the Akaike Information Criterion corrected (AICc^70,71^) obtained for each combination of rm and fc. Secondarily, the performance and realism of the models was supervised inspecting the values of omission rate under the 10-percentile threshold (OR 10), the area under the curve (avg AUC) values, the estimated response curves^72,73^, and the potential geographical distributions obtained after the spatial projection of each niche model. Constructed final niche models were spatially projected onto a study region that encompasses the geographic distribution of *Abrothrix olivacea* (Supplementary material 2). During transferences of models, environmental differences between training and projection climate scenarios were assessed by performing Multivariate Environmental Similarity Surface (MESS; see^74^) analysis, to determining the spatial distribution of analogous or non-analogous climate conditions between the climate scenarios considered in each model transference instance. These procedures were completed using the *ntbox* R package^75^.

Following indications of Guevara et al.^76^, niche model transferences were completed using an unconstrained extrapolation. The past climatic scenarios explored corresponded to the reconstructions performed by Otto-Bliesner et al.^77^ for climatic conditions of the LIG, Watanabe et al.^78^ for climatic conditions of the LGM and the Mid-Holo, and Hijmans et al.^46^ for the Current climatic conditions. Finally, the geographic representations of the climatically suitable areas were constructed using a continuous representation of environmental suitability values, which were spatially projected using QGIS.

## Results

### Mitochondrial data and genetic structure

**Figure 1:**
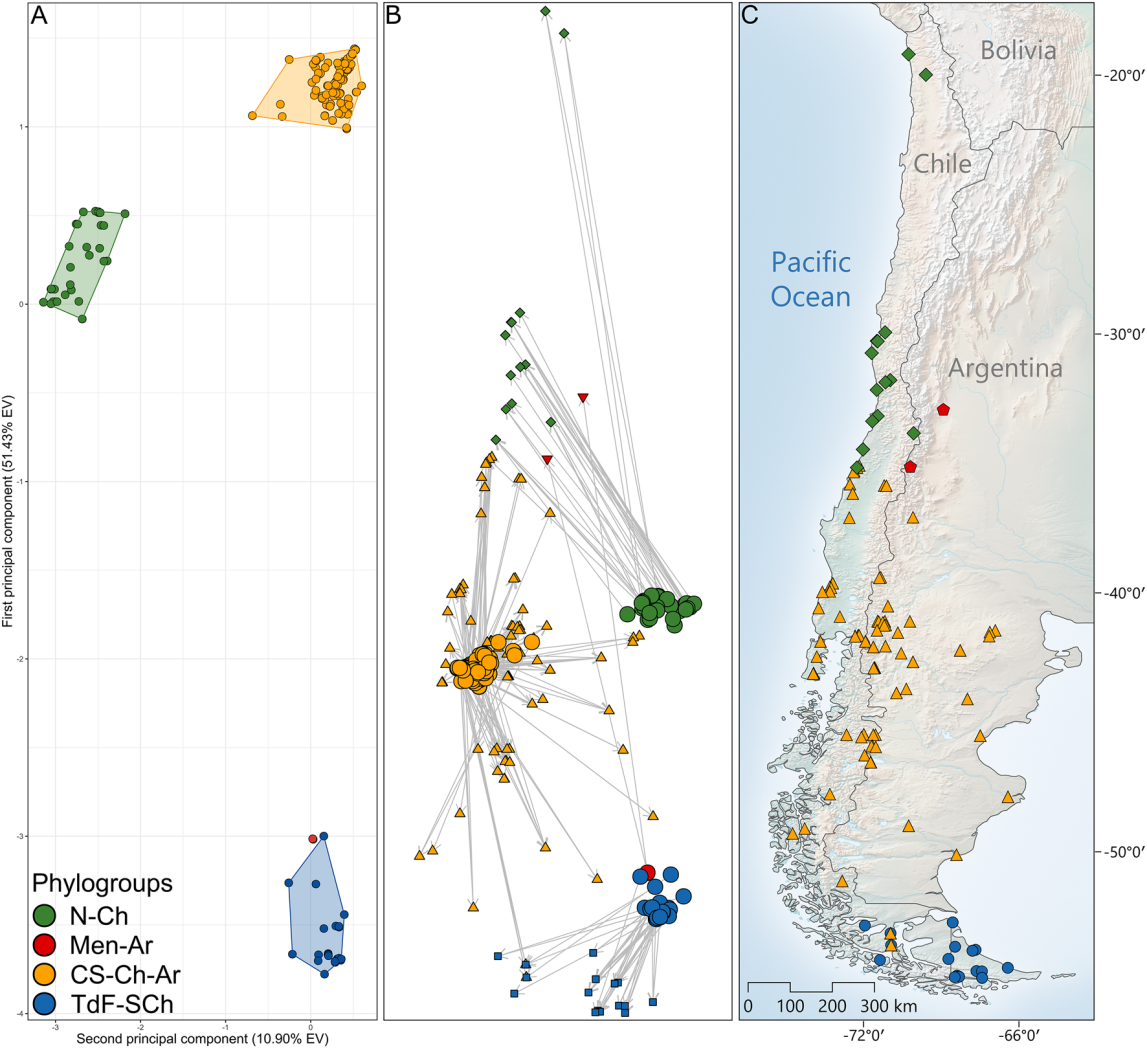
Procrustes analysis of the gPCA of *Abrothrix olivacea* and the 103 localities where individuals were captured. (**A**) Genetic map constructed from the projection of the individuals onto the orthogonal intersection of the two first principal components of the gPCA (SM, Fig. S3). (**B**) Procrustes superimposition of the transformed maps showing the position of individuals on the genetic map (larger circles) and on the geographic map (green diamonds = N-Ch, yellow triangles = CS-Ch-Ar, red inverted triangles = Men-Ar, and blue squares = TdF-SCg). The length of the line connecting individuals in gPCA map to their geographical location represents the extent of the deviation from the expected pattern of genetic variation based on geography (see Knowles et al.^77^). (**C**) Geographic map constructed from geographical coordinates of the localities where the individuals were sampled; this plate was composed using QGIS, version 3.18.2-Zürich. Localities are colored according to colors gave to the mitochondrial phylogroups.

186 mt-SNPs were identified from the alignment *Cytb* sequences of *Abrothrix olivacea*. The gPCA performed with this dataset shows complete differentiation among the four mitochondrial phylogroups (Fig. 1A; SM, Fig. S3). This gPCA was completed retaining the first 20 gPCs which explain the 90.74% of the total variance, and from these, the first three gPCs account for the 69.79% of the total variance (gPC1 = 51.44%, gPC2 = 10.91%, gPC3 = 7.45%).

### Spatial-climatic correlations and landscape genetics analyses

Just six of the 19 bioclimatic variables using in the climatic characterization were not correlated with each other: Bio 03: isothermality ([Bio 02/Bio 07] × 100), Bio 04: temperature seasonality, Bio 06: min temperature of coldest month, Bio 09: mean temperature of driest quarter, Bio 15: precipitation seasonality, and Bio 16: precipitation of wettest quarter (SM, Table S1). In addition, all Mantel tests performed with the matrices of geographic distancing and climatic dissimilarity indicate significative association (i.e., *p-*value < 0.05); both, for the whole sample of *Abrothrix olivacea* (*r* = 0.59, *p* = 0.00) and mitochondrial phylogroups (N-Ch: *r* = 0.53, *p* = 0.00; CS-Ch-Ar: *r* = 0.54, *p* = 0.00; TdF-SCh: *r* = 0.44, *p* = 0.00). IBD and IBE evaluations also resulted with significant values of correlations at the spatial scale corresponding to the geographic range of *A*. *olivacea* (Table 1). When these models were evaluated for the phylogroup geographic, only significant correlations were found for N-Ch (IBD: *r* = 0.282, *p*-value = 0.041) and TdF-SCh (IBE: *r* = 0.346, *p*-value = 0.047) (Table 1; SM, Figs. S4 and S5). In turns, partial Mantel test indicate significant correlation only for the dataset corresponding to the N-Ch phylogroup (Table 1).

**Table 1:** Results of Mantel tests and partial Mantel tests performed to evaluate models of isolation by distance (IBD) and isolation by environment (IBE) in *Abrothrix olivacea* and their mitochondrial phylogroups (N-Ch, CS-Ch-Ar, TdF-SCh; see text).

Genetic dataset	Isolation by distance		Isolation by environment			
	Mantel test		Mantel test		Partial Mantel test	
	*r*	*p-*value	*r*	*p-*value	*r*	*p-*value
*A. olivacea*	0.065	0.026*	0.066	0.018*	0.034	0.132
N-Ch	0.282	0.041*	0.025	0.363	0.230	0.046*
CS-Ch-Ar	0.049	0.146	0.053	0.159	0.024	0.296
TdF-SCh	0.056	0.334	0.346	0.047*	− 0.147	0.849

For each model evaluation values of Mantel statistic based on Pearson's product-moment correlation index (*r*) and its significance (*p*-value) are presented. Significant values (*p*-value ≤ 0.05) are indicated with an asterisk.

The PA indicates the existence of an optimal alignment between the coordinates projected in the genetic map with the coordinates projected in the geographic map, which implies a rotation of 36.49° in the counterclockwise direction of the orthogonal projection of the genetic PCs (Fig. 1). In turn, the resemblance of these maps was indicated by a strongly statistically supported Procrustes similarity score (*t*_*0*_ = 0.637; *p-*value = 1 × 10^–4^; SM, Fig. S6). The plot of the superimposed maps depicts that the genetic differences of the populations are geographically structured and exhibit different magnitudes along the geographic distribution of *Abrothrix olivacea*. The lines connecting individuals from the gPCA map to their geographical locations have lengths that are significantly different among the mitochondrial phylogroups (ANOVA *p-*value = 1 × 10^–4^; SM, Fig. S6), being longer among N-Ch localities and shorter among CS-Ch-Ar and TdF-SCh localities. The length of the line connecting individuals in gPCA map to their geographical location represents the extent of the deviation from the expected pattern of genetic variation based on geography, see^79^.

**Figure 2:**
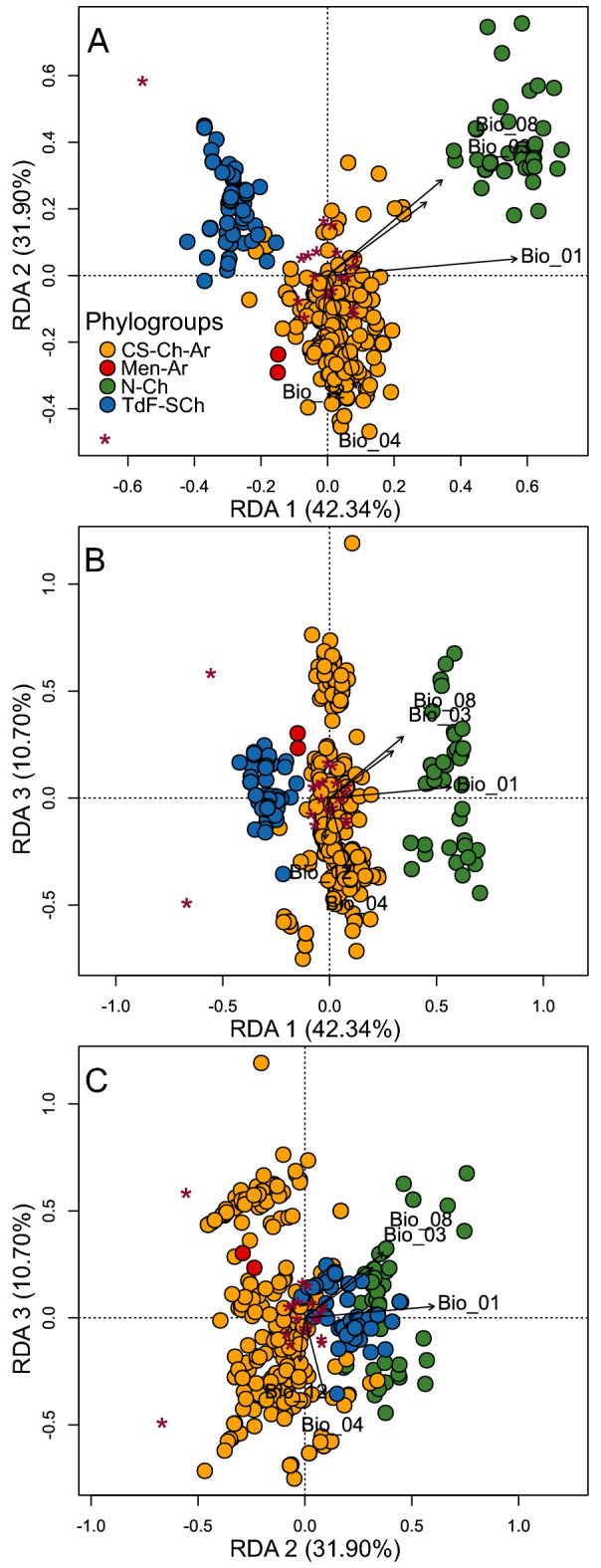
Triplot showing the climatic variables that best explain the pattern of mitochondrial genetic structure of *Abrothrix olivacea* implementing a Partial Redundancy Analysis (RDA) conditioned by geographic distance. Sampled individuals are represented by the circles, which are colored according to their correspondence to each mitochondrial phylogroup (see legend). Red asterisks represent the scores of the first 20 principal components of the gPCA that were employed as response variables. Arrows correspond to the variables that were significant (*p*-value ≤ 0.05) for the analysis, according to results obtained with the ordistep function (see Table 5); their length indicates the magnitude of their explicative contribution, and the angle reflects the correlation among them. From (**A**–**C**), the orthogonal projections of the first three RDA axes, which cumulated 84.93% of explained variance, are shown (see Table 3).

The RDA shows that 8.17% (*R*^2^ adjusted = 0.0817) of the total mitochondrial genetic variance is explained by the geographical distancing of the localities, while 87.78% (*R*^2^ adjusted = 0.8778) is explained by the climatic variables included as model predictors. Among these, only five (Bio 01: Annual Mean Temperature, Bio 03: Isothermality (Bio 02/Bio 07) (× 100)), Bio 04: (Temperature Seasonality (standard deviation × 100)), Bio 08: Mean Temperature of Wettest Quarter, and Bio 12: Annual Precipitation; Table 2) significatively explain (*p-*value ≤ 0.05) the pattern of mitochondrial genetic structure exhibited by *Abrothrix olivacea*. Together, these climatic predictors accumulate 53% of the variance (i.e., a proportion of constrained inertia of 4.01 from a total inertia of 7.54) after removing the variance explained by geography, while geographic distance solely accounted for 8.61% (conditioned inertia = 0.61) after removing the variance due to climatic heterogeneity. Finally, climate and geography together have a combined effect of 38.34% (unconstrained inertia = 2.89) on genetic variance, which is the amount of variance in which due to collinearity, climate and geography cannot be separated^80^. The first three axes of the RDA accounted for 84.93% of the constrained genetic variation, and Bio 01, Bio 03 and Bio 08 were the variables with higher explanatory contribution in the RDA model (Table 3). These predictors are positively correlated with the pattern of genetic structure, as demonstrated their position in the RDA axes (Fig. 2). Conversely, the position of the Bio 04 and Bio 12 along the RDA 2 and RDA 3 axes indicate that these variables are negatively correlated with the genetic variation accounted by the first 20 PCs retained from the gPCA. In turns, the scattering of the individuals on the orthogonal projection of the RDA axes shows that each mitochondrial phylogroup have mostly nonoverlapped distributions that are differently associated with climatic predictors, depicting that the different levels of internal genetic structure of these clusters are associated with different climatic configurations.

**Table 2:** Climatic variables that, according to stepwise model selection performed to complete the RDA, significantly explain the genetic structure pattern of *Abrothrix olivacea*.

Bioclimatic variables	AIC	F	*p-*value
Bio 01: annual mean temperature	657.61	60.3811	0.005
Bio 03: isothermality (BIO2/BIO7) (× 100)	608.24	7.9623	0.005
Bio 04: temperature seasonality (standard deviation × 100)	651.10	53.1093	0.005
Bio 08: mean temperature of wettest quarter	604.06	3.7997	0.010
Bio 12: annual precipitation	649.68	51.5342	0.005

Values of the Akaike Information Criterion (AIC), F statistics (F), and statistical significance (*p-*values) are presented only for the uncorrelated bioclimatic variables.

**Table 3:** Results of partial redundance analysis (RDA) performed to evaluate the association between the pattern of mitochondrial genetic structure of *Abrothrix olivacea* and the climatic variability of its geographic distribution.

gPCs	RDA1	RDA2	RDA3
PC 1	− 1.089	1.065	0.097
PC 2	− 1.310	− 0.895	− 0.092
PC 3	− 0.004	− 0.113	− 0.145
PC 4	0.164	0.098	− 0.295
PC 5	− 0.076	− 0.008	0.014
PC 6	0.080	− 0.005	− 0.395
PC 7	− 0.064	0.132	0.017
PC 8	− 0.137	− 0.230	0.169
PC 9	− 0.154	0.095	− 0.079
PC 10	0.156	− 0.214	0.510
PC 11	0.029	− 0.083	0.004
PC 12	0.152	− 0.182	0.000
PC 13	− 0.171	− 0.139	− 0.264
PC 14	0.115	− 0.009	− 0.038
PC 15	0.056	0.125	0.085
PC 16	0.148	0.050	− 0.198
PC 17	− 0.008	− 0.087	0.168
PC 18	0.026	0.275	− 0.238
PC 19	− 0.023	0.299	0.042
PC 20	− 0.115	0.110	0.073
Eigenvalue	0.684	0.515	0.173
% of explained variance	42.349	31.892	10.692
F	15.502	11.674	3.914
Axis significance (*p-*value)	0.001	0.001	0.001

The loadings of the 20 first principal component (gPCs) obtained from the genetic based PCA on the three first RDA axes are presented. In addition, the eigenvalue, explained variance, F value and the significance (*p-*value) of each RDA axis is shown.

### Climatic niche differentiation and spatial predictions of climatic suitability

**Figure 3:**
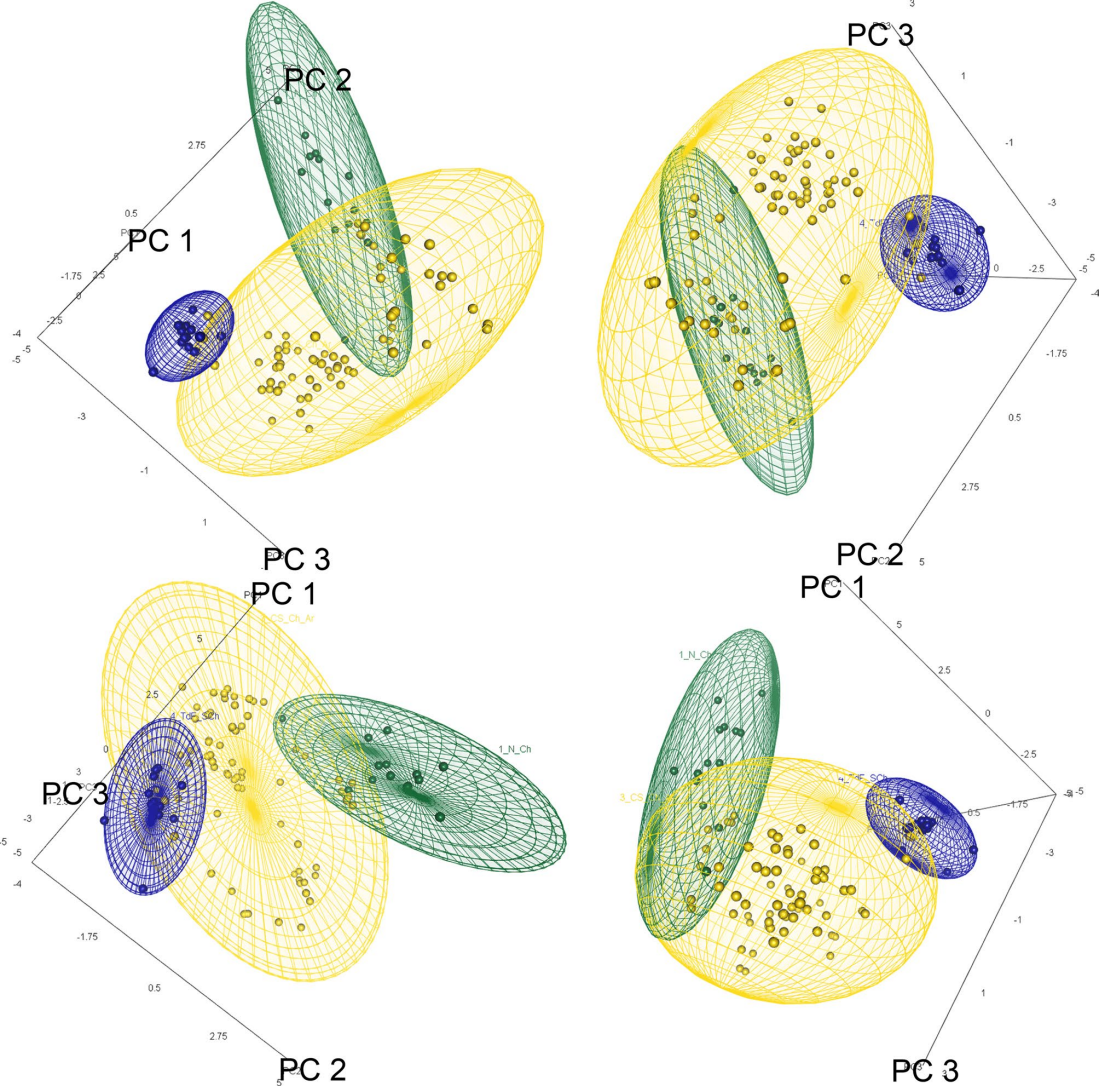
Four views of the climatic space constructed with the orthogonal projection of the first three principal components obtained from the PCA based on the climatic characterizations of the geographic ranges of the mitochondrial phylogroups of *Abrothrix olivacea*. The climatic hypervolume of each phylogroup is represented by each ellipsoid, and these are colored according to colors given to each phylogroup in the Fig. 1. The phylogroup from Mendoza, Argentina (Men-Ar) was not represented because at least three points are needed for the construction of a tridimensional ellipsoid.

Unambiguous differences in breadth and composition among the climatic spaces occupied by each mitochondrial phylogroup were evidenced with the PCA performed based on the climatic characterizations (MS, Fig. S7). The first three PCs of this analysis explained 88.97% of the variance (MS, Table S2). The differences of the pairwise comparisons of the climatic attributes of the geographic ranges of each mitochondrial phylogroup are significant (*p*-value ≤ 0.05), except for the comparison between CS-Ch-Ar and Men-Ar (Table 4). In addition, notably differences among the sizes of the climatic hypervolumes were identified (TdF-SCh = 13.54 vs. N-Ch = 101.92 vs. CS-Ch-Ar = 447.68), as well as in their placement in the environmental space (Fig. 3), having low levels of overlap among them (Table 5).

**Table 4:** Results of the pairwise comparisons between the climatic niche of each mitochondrial phylogroup of *Abrothrix olivacea* assessed with a Permutational Multivariate Analysis of Variance (PERMANOVA).

	N-Ch	Men-Ar	CS-Ch-Ar
Men-Ar	0.017*		
CS-Ch-Ar	0.000*	0.096	
TdF-SCh	0.000*	0.005*	0.000*

Significant differences (*p*-value ≤ 0.05) between are indicated with an asterisk.

**Table 5:** Indexes of overlap and uniqueness of the climatic hypervolumes of the mitochondrial phylogroups of *Abrothrix olivacea*.

Pair of mitochondrial phylogroups	Climatic niche overlap		Climatic niche uniqueness	
	Jaccard	Sørensen	Unique fraction of 1st phylogroup	Unique fraction of 2nd phylogroup
N-Ch vs. CS-Ch-Ar	0.085	0.157	0.576	0.904
N-Ch vs. TdF-SCh	0.000	0.000	1.000	1.000
CS-Ch-Ar vs. TdF-SCh	0.026	0.051	0.974	0.143

The calculated overlap for the compared phylogroup pairs and their uniqueness are provided. Values can range from 0 to 1. The phylogroup from Mendoza, Argentina was not included in these analyzes.

After spatial filtering, 364 presence records of those 4925 retrieved from GBIF were retained to develop the niche models of *Abrothrix olivacea*; of these, 24, 67, and 273 were assigned to the TdF-SCh, N-Ch, and CS-Ch-Ar phylogroups, respectively. The best configuration for constructing the niche model for *A*. *olivacea* was a combination of linear as fc and 1.5 as the value for rm, while for the mitochondrial phylogroups the configurations indicated as ‘best’ in the Table S3 (see SM) where employed. In general, these model configurations showed good performance, as indicated by AUC and omission rate values higher than 0.85 and lower than 0.10, respectively.

**Figure 4:**
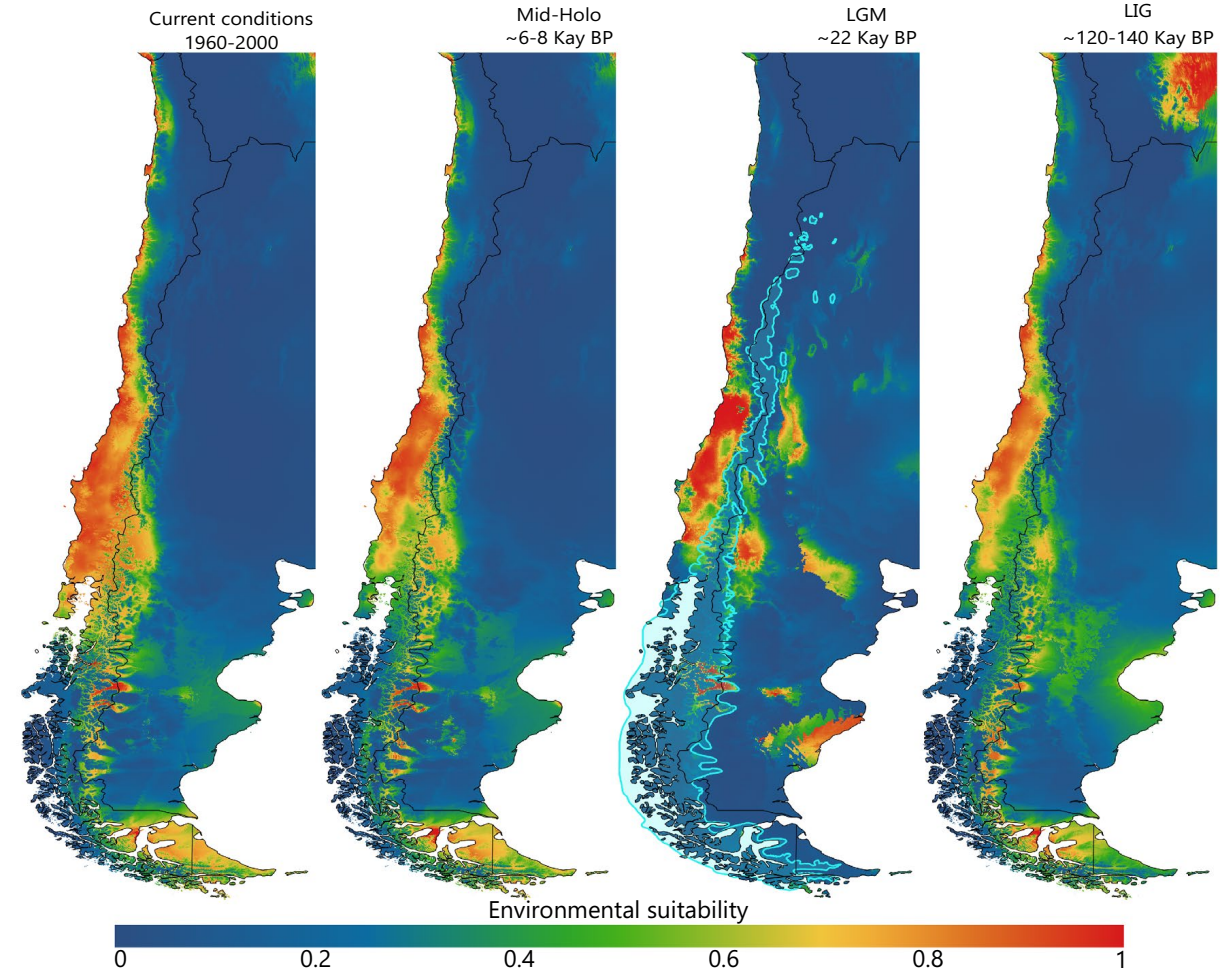
Potential geographic distribution of the climatically suitable areas for *Abrothrix olivacea* onto the climatic scenarios explored from present to past (Current conditions: 1960–1990; Mid-Holocene: ∼6–8 Kay BP; Last Glacial Maximum: ∼22 Kay BP; Last Interglacial: ∼120–140 Kay BP). Each panel depicts the spatial projection of the climatic niche model constructed for this species in the climatic scenarios explored. The light blue shading represents the extent of the Patagonian Ice Sheet during the LGM according to McCulloch et al.^112^. Climatic-Environmental suitability values are depicted as continuous representation and areas with warmer colors indicate regions whit higher climatic suitability.

**Figure 5:**
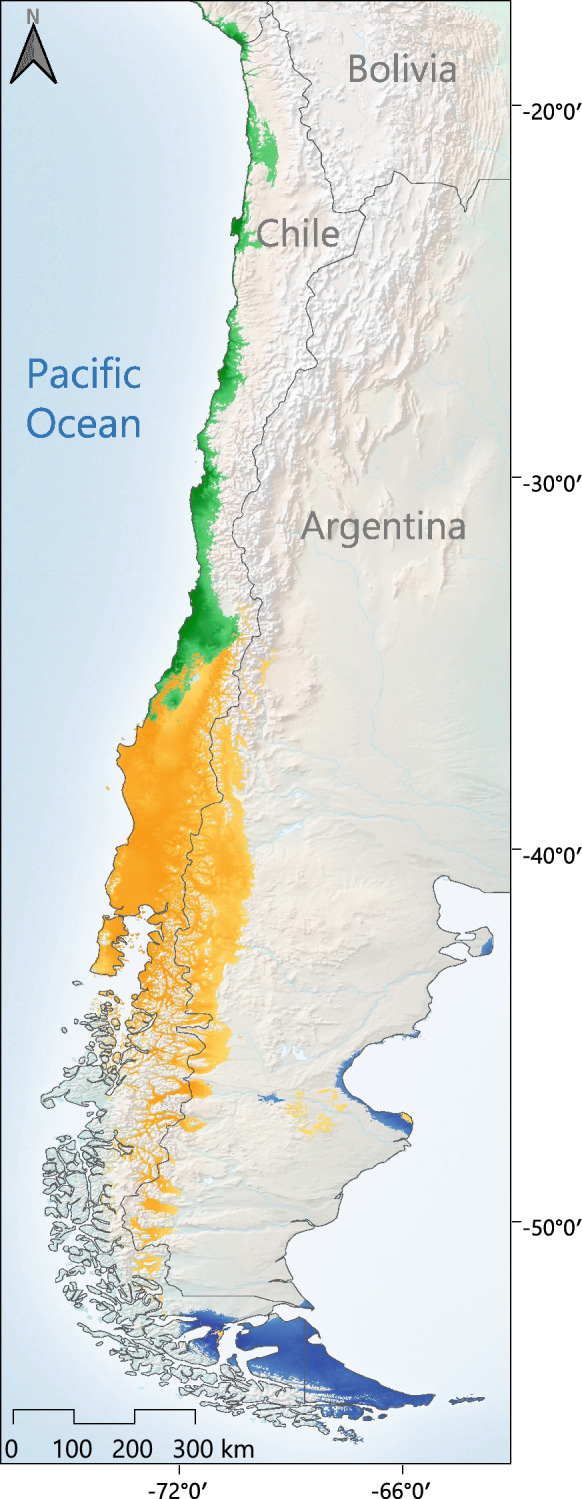
Potential geographic distributions of three of the mitochondrial phylogroups of *Abrothrix olivacea* as estimated from the projection of the upper values (0.55–1) of climatic suitability in the Current climatic conditions (1960–1990). Colored shading areas represent the climatically suitable areas estimated for the phylogroups N-Ch (green), CS-Ch-Ar (yellow), and TdF-SCh (blue), and the intensely colored zones correspond to those where the climatic conditions are optimal for each phylogroup.

The spatial projection of the niche models onto the climatic scenarios explored showed that the geographic distribution of the suitable climatic conditions for *Abrothrix olivacea* have undergone changes from the LIG to the Present (Fig. 4). The major area reductions were evidenced by the projection of the niche models onto the reconstruction of the LGM climatic condition and mostly involve areas that were covered by the Patagonian Ice Sheet during this climatic episode (SM, Fig. S8). In the case of the mitochondrial phylogroups, the spatial projection of their niche models shows that the suitable climatic condition remained almost constant for all. The only remarkable reductions of the climatically suitable areas were observed during the climatic conditions of the LGM; specifically in the southern portion of the geographic distribution of the CS-Ch-Ar and the entire region where the TdF-SCh is currently distributed (SM, Fig. S9). In this regard, the MESS analyses indicated that the areas with non-analogous environmental conditions in the explored past climate scenarios were small, and mostly associated with regions that correspond to areas outside the observed geographic distribution of the species and/or mitochondrial phylogroups considered (SM, Fig. S10); therefore, the uncertainty associated to model transfer is minor. Meanwhile, for this last phylogroup, the LIG was the historical episode that offered the least restrictive climatic conditions. In addition, the simultaneous spatial projection of the niche models of the mitochondrial phylogroups onto the climatic scenario corresponding to the Current climatic conditions, demonstrated that the potential geographic distributions of their climatically suitable areas are largely disjunct across the geographic space of southern South America (Fig. 5).

## Discussion

The multivariate analyses of mitochondrial variation, climatic characterizations and geographic coordinates evidenced that the pattern of genetic structure exhibited by *Abrothrix olivacea* is associated to the geographical deployments of their populations and the climatic heterogeneity of the landscapes in which they occur. In particular, the evaluations focused on the mitochondrial phylogroups demonstrated that strength of these genetic–climate/geography correlations differ throughout the geographic distribution of this species, and hence among their mitochondrial lineages. Certainly, it should not be forgotten that genetic assessments based on a single locus or few loci present limitations, and that the suitability of mtDNA in evaluations such as performed in this study have been questioned^81,82^. Nevertheless, it is obvious that all genetic datasets have restrictions, so results should be always interpreted with caution, e.g.^83^. Beyond this, the assessment presented here should be considered as a first step useful to advance hypotheses that could be tested using a genomic approach.

This study represents the first assessment based on multivariate analyses, correlative niche models, and landscape genetics approaches in evaluate whether the structure of the mitochondrial variability of *Abrothrix olivacea* correlates with climatic and spatial features of the South American landscape. As was already mentioned, the patterns of mitochondrial genetic structure exhibited by populations of *A. olivacea* had been studied based on different geographic coverage and focusing on different populations, e.g.^33,40,41,84–86^, but none of these had contextualize the uncovered patterns of genetic variation with explicit spatial analyses. The relevance of the climatic dynamism that occurred from the Quaternary to the Present in shaping the patterns of geographic and environmental distribution exhibited by *A. olivacea* as well as its main lineages is evidenced here. Overall, by providing clues as to how intraspecific pattern of mitochondrial genetic structure would have originated and differentiated, these findings help to better understand the evolutionary history of *A*. *olivacea*.

### Landscape associations of the patterns of mitochondrial structure

The spatially and climatically explicit assessment of the mitochondrial variability of *Abrothrix olivacea* indicates that the patterns of genetic structure are influenced by landscape features and are consistent with the predictions of IBD and IBE models (Table 1). In this regard, both implementations of the Mantel tests based on climatic and geographic distances indicated that climatic differentiation of the genetic-sampled localities increases according to their geographic separation. This implies that the forces imposed by geographic distances operate in association to climatic dissimilarities to also generate the pattern of genetic structure observed within mitochondrial phylogroups of *A*. *olivacea*, and probably distinguishing their individual effects may not be straightforward. In addition, results of the PA indicated that the effect of geographic distancing on the genetic differentiation of populations is comparatively greater in the populations from northern Chile, respect those central and southern distributed (Fig. 1); which in turn suggests that individual dispersion is more disrupted by geographic distancing in the arid environments of northern Chile, as indicated the major deviations from the expected pattern of genetic variation based on geography exhibited by populations occurring along this region (SM, Fig. S6). These latter results are consistent with those obtained with the RDA, which in addition to indicate that the climatic features considered are significatively associated to the pattern of genetic structure, also evidenced that warmer and dryer climatic conditions (e.g., such as those present along northern Chile) are associated with higher levels of genetic differentiation; while in colder regions with higher rainfalls (e.g., western Patagonia and southern Tierra del Fuego) genetic differences are lower (Fig. 2).

Differences among the internal patterns of genetic structure exhibited by each mitochondrial phylogroup of *Abrothrix olivacea* suggest that the geographic distancing and the climatic dissimilarity heterogeneously influence the dispersal abilities of individuals throughout the species geographic distribution. For example, the Mantel Tests and the Partial Mantel Tests (Table 4) consistently indicated that structuring of the mtDNA variation in the N-Ch phylogroup might fit to the model of strict IBD^23^, since genetic differences appear not be significantly correlated to the climatic dissimilarities among localities (Table 1; SM, Figs. S4 and S5). In arid environments from northern and central Chile (e.g., Coastal desert, Mediterranean shrub), extremely scarce rainfalls and soil characteristics cause clumps of vegetation scattered along a matrix of bare soil, where areas covered by vegetation and those exposed-soil areas have strongly sharp climatic differences^87–90^. In structured arid landscapes, small mammal species live mostly confined within patches of vegetation and/or in locations where these patches are more densely arrayed^91,92^; this fact implies that in such environmental conditions these animals tend to limit their areas of action and therefore, their home ranges. So, it is feasible that genetic differences among these populations could be actually caused by the geographic separation of the suitable habitat patches in which restrictively individuals inhabit, and not by the independent effect of the geographic distancing^93^. In addition, it is also feasible that changes in vegetation coverture caused by seasonal fluctuations in climatic conditions can directly affect the vagility of individuals at these regions, altering as such the gene flow among populations. Beyond these plausible explanations, the fact that the Mantel test does not detect the proportion of the genetic structure determined by the climatic heterogeneity within the N-Ch phylogroup (as already indicate the RDA results) could also simply be due to the inherent limitations of this analysis (see^94^). For instance, our data and analyses do not allow assessing the effect of seasonality and topographic variation on home range changes and of the latter in the geographic structure of the genetic variation.

On the contrary, the levels of internal genetic structure exhibited by the mitochondrial phylogroups CS-Ch-Ar and TdF-SCh, distributed along central and southern Chile and Argentina, are comparatively low^37^, are not correlated to geographical distances, and significant association with climatic dissimilarity was only evidenced for the phylogroup distributed in southernmost Chile and Tierra del Fuego Island (i.e., TdF-SCh; Table 1; SM, Figs. S4 and S5). This specific result is striking because considering the positive correlation that was determined between geographic distances and climatic dissimilarities, a significant IBD pattern was expected. However, as this region is bisected by marked climatic differences take place at a narrow strip (i.e., exist more climatic heterogeneity per unit of area, respect to Patagonia and northern Chile), the directionality of genetic distances may be disposed following the array of environmental dissimilarities. Be that as it may, the IBE pattern observed in this lineage does not strictly imply a causality relationship between these aspects. On the contrary, this pattern of correlation might have other but more complex explanations (e.g., genetic co-adaptation, genetic draft; see^95,96^, that cannot be explicitly assessed with the data and methodology employed in this study. Thus, future characterizations of the genetic variation of this species should consider the outlined limitations and include the analysis of the variation of several candidate genes or specific regions of the genome^97^, which would allow elucidating the potential effect of natural selection imposed by Patagonian environmental heterogeneity in driving and shaping populational divergence within this species. Similarly, a more geographically dense sampling should be conducted to further assess the geographic distribution of the distinct phylogroups; in particular that of the one so far restricted to the Argentinean province of Mendoza (i.e., Men-Ar). Additionally, the combination of broader geographic and genetic sampling would allow test the relationships of the phylogroups and localities that so far have been only characterized via mitochondrial variation.

### Niche differentiation and spatial dynamics of climatic suitability

The climatic differences among the geographic ranges of the mitochondrial lineages of *Abrothrix olivacea* revealed that their realized climatic niches are markedly different. The results obtained with PCA (Fig. S7, Table S2), PERMANOVA (Table 5), and the construction and overlapping of Gaussian hypervolumes in the constructed environmental-climatic space (Fig. 5, Table 5) demonstrated the existence of measurable and significant differences in terms of constitution and occupancy of climatic variables among the compared mitochondrial lineages. The climatic niche of a species is constituted by all climatic conditions that exist across its geographic distribution^98,99^. In turn, the portion of these conditions that is actually occupied by the individuals, once biological interactions have taken place, represents its realized ‘climatic’ niche^61,100^. This ecological entity is embedded in the potential ‘climatic’ niche (i.e., scenopoetic fundamental niche^61^); a species’ property that is determined primarily by its physiological capabilities^101,102^ and whose manifestation in the environmental space represents the maximum possible expression of its ecology^61^. The potential ‘climatic’ niche is critically important since it largely determines where a species can occur, as well as how it has or will be able to respond to past and upcoming climatic changes^103,104^. Thus, the differences observed among the climatic niches of the mitochondrial lineages of *A. olivacea* are in terms of their realized climatic niches, since the comparisons performed are based on climatic attributes characterized at the localities where these lineages occur (i.e., observed geographic distribution^61^). This clarification is important because although the observed differences indicate that the realized climatic niches of these lineages are mostly distinct, these differences could be different expressions of the species potential climatic niche.

The absence of geographic reciprocity among the spatial projections of the niche models of each mitochondrial lineage (Fig. 5), evidence that niche differentiation not only manifests along the climatic dimensions of the ecological niche, but also across the geographic space where lineages distribute. This result suggests that each lineage is geographically restricted to different climatic regimens, where, as a consequence, its individuals will interact with different biotic elements^97,105^. Geographically structured variation of biological traits, as the climatic niche, could represent environmentally-induced plastic responses^12^ or, alternatively, local adaptations to specific climatic conditions or other environmental aspects^106,107^. Particularly for *Abrothrix olivacea*, given evidence for population differences in physiological traits^108–111^ and levels of genetic expression^97^ among populations distributed in disparate environmental conditions have been described. The spatial differences detected are overlooked if visual inspections are limited to the projection of the niche model developed at the species level (Fig. 4) but are noticeable when the niche model of each lineage are simultaneously projected (Fig. 5); these are also seen when the niche models are projected through the explored past climatic scenarios (SM, Fig. S9). This emphasizes the importance of considering intraspecific variation when modeling the ecological niche of a species with phylogeographic structure and/or a wide geographic distribution^58,112,113^. Together, the outlined results suggest that the climatic niches of these lineages show some degree of divergence, although a priori this should not be expected among intraspecific lineages, given that some level of gene flow must exist among them^6,104^. However, some degree of variation in climatic preferences may be exhibited among intraspecific lineages, but in principle, these should fit within the breadth of the species’ fundamental niche^99,114^. Future assessments directed to understand this observation could be carried out through coupling physiological experiments and mechanistic niche models^101^.

Projections of the developed niche models onto the explored past climatic scenarios, which did not involve extrapolation to non-analogous climatic conditions (SM, Fig. S10), suggest that the climatic changes occurred from the LIG to the Present driven important spatial dynamics of the climatically suitable areas of *Abrothrix olivacea* and its mitochondrial lineages (Fig. 4; SM, Fig. S9). Specifically, these estimations demonstrated that glaciations did not affect populations in northern Chile; on the contrary, populations distributed in western Patagonia and Tierra del Fuego were largely affected and as their climatically suitable areas were significatively reduced. These reconstructions are realistic given that there is evidence that glaciations were less intense towards the northern fraction of the species range^115,116^, while the ice sheet largely extended across southern latitudes below ca. − 37°, covering a large area of Patagonia and Tierra del Fuego^117^. The effects of the Patagonian glaciations on small mammal species of southern South America have been addressed by several studies^30,33–36^, and in most cases the population size reductions that were inferred were associated to contraction of species ranges due to the advance of the ice sheet. Reconstructions of the historical demography of *A*. *olivacea* and their lineages based on mitochondrial variability were recently performed and indicate that Patagonian and Fueguian populations have undergone expansions that predates the LGM (around 70 Kay BP in populations from continental Patagonia [CS-Ch-Ar], and 55 Kay BP in those distributed in the Tierra del Fuego Island and surrounding continental regions [TdF-SCh]); see^37^. Meanwhile, demographic inferences based on nuclear genomic variation, but with a narrower geographic and specimen sampling, indicate that Patagonian populations expanded more recently (around 13 Kay BP), and likely in response to the retreat of the ice sheet at the end of the LGM^118^. This pattern is consistent with the expansion of climatically suitable areas as inferred with the model projections in the climatic scenarios corresponding to the LGM and the Mid-Holocene (SM, Figs. S8 and S9). The coupling of reconstructions of past geographic distributions made with ENM and estimates of demographic histories has been widely used to assess the importance of events such as glaciations on processes associated with fluctuations in genetic variation^119,120^, see^36^ for an example of the usage of this approach on *Abrothrix longipilis*; and particularly for *A*. *olivacea*, it is shown that it served to better pondered the effect of climatic events on the differentiation of its populations. Finally, a note of caution is needed regarding the paleodistributional changes here advanced, as they largely depend on the used General Circulation Model; depending on the case, differences among estimates would be more or less extensive depending on the uniqueness, resemblance, or dissimilarity of the climate scenario where the model was created and the scenario where it is being transferred; see^121,122^.

## Concluding remarks

The results of this study highlight patterns that will be relevant for the design of future studies aimed to address aspects related to ecological influenced genetic differentiation. On the one hand, it is important to consider that given the complex geological evolution and the large environmental heterogeneity exhibited across southern South American, patterns of genetic structure and/or ecological niche differentiation discovered in species from this region would likely be complex, and their complete unraveling will require samplings that exhaustively characterize the intraspecific genetic and ecological variation. This does not imply that all South American species have complex evolutionary histories, but potentially all those with deep phylogeographic breaks, large geographic distributions and inhabiting distinct landscape configurations as *Abrothrix olivacea* (e.g., *A*. *hirta*, *Loxodontomys micropus*, *Oligoryzomys longicaudatus*; see^123^ for a recent exploration in this line), would likely do. Similarly, codistributed species with large phenotypic variation (e.g., *A. hirta*; see^124^) are worth of analysis. Thus, future research strategies focused on these species should contemplate the possibility that different evolutionary mechanisms are acting with uneven intensities on their populations, requiring the development of methodologies and approaches that cover distinct populations simultaneously and that, in turn, consider the heterogeneity of the landscape in which they exist. A good starting point under this premise could be achieved by using genetic data generated by previous studies but analyzing them with the approaches and methodologies used here, see^80,125^, ideally those containing comprehensive and exhaustive genetic samplings^34,118^. In this line, the characterization and analysis of the dimensions that constitute the ecological niche can become a mandatory procedure when attempting to study the dynamics of species with significant levels of population differentiation^58,126^. This is important because populations or lineages within a species could have different evolutionary histories, shaped for example, by geophysical processes occurring locally, whose effect can be overlooked if ecological attributes characterized are only analyzed at the species level.

## Supplementary Information

Supplementary Information.

## Data Availability

All DNA sequences employed in this study are already deposited in GenBank; accession numbers are listed in Appendix 1.
