# Supplementary material for: Climate influences the genetic structure and niche differentiation among populations of the olive field mouse Abrothrix olivacea (Cricetidae: Abrotrichini)
Source: Sci Rep. 2022 Dec 27;12:22395. doi: 10.1038/s41598-022-26937-x (PMC9794701; doi:10.1038/s41598-022-26937-x)

**SUPPLEMENTARY MATERIALS**

**CLIMATE INFLUENCES THE GENETIC STRUCTURE AND NICHE DIFFERENTIATION AMONG POPULATIONS OF THE OLIVE FIELD MOUSE *ABROTHRIX OLIVACEA* (CRICETIDAE: ABROTRICHINI)**

Marcial Quiroga-Carmona and Guillermo D’Elía

**Appendix 1.** GenBank accession numbers of the 416 Cytb sequences of *Abrothrix olivacea* employed in the analyses of genetic structure and landscape genetics. Sequences are grouped according to the mitochondrial phylogroups described by Quiroga-Carmona et al. (2022). Geographic coordinates and numeration of the localities in the map showed in Figure S1.

| GenBank code | *Cytb* phylogroup | Locality number | Latitude | Longitude |
| --- | --- | --- | --- | --- |
| MW890327 | CS-Ch-Ar | 15 | -35.081 | -72.167 |
| MW890328 | CS-Ch-Ar | 15 | -35.081 | -72.167 |
| MW890329 | CS-Ch-Ar | 15 | -35.081 | -72.167 |
| MW890330 | CS-Ch-Ar | 15 | -35.081 | -72.167 |
| MW890331 | CS-Ch-Ar | 15 | -35.081 | -72.167 |
| MW890332 | CS-Ch-Ar | 15 | -35.081 | -72.167 |
| MW890333 | CS-Ch-Ar | 15 | -35.081 | -72.167 |
| MW890334 | CS-Ch-Ar | 15 | -35.081 | -72.167 |
| MW890335 | CS-Ch-Ar | 15 | -35.081 | -72.167 |
| MW890336 | CS-Ch-Ar | 15 | -35.081 | -72.167 |
| MW890337 | CS-Ch-Ar | 15 | -35.081 | -72.167 |
| MW890310 | CS-Ch-Ar | 17 | -35.161 | -72.254 |
| MW890313 | CS-Ch-Ar | 17 | -35.161 | -72.254 |
| MW890316 | CS-Ch-Ar | 17 | -35.161 | -72.254 |
| MW890315 | CS-Ch-Ar | 18 | -35.288 | -72.365 |
| MW890314 | CS-Ch-Ar | 19 | -35.335 | -72.386 |
| MW890392 | CS-Ch-Ar | 20 | -35.802 | -72.531 |
| MW890393 | CS-Ch-Ar | 20 | -35.802 | -72.531 |
| MW890394 | CS-Ch-Ar | 20 | -35.802 | -72.531 |
| MW890395 | CS-Ch-Ar | 20 | -35.802 | -72.531 |
| MW890396 | CS-Ch-Ar | 20 | -35.802 | -72.531 |
| MW890397 | CS-Ch-Ar | 20 | -35.802 | -72.531 |
| MW890398 | CS-Ch-Ar | 20 | -35.802 | -72.53 |
| MW890305 | CS-Ch-Ar | 21 | -35.856 | -71.204 |
| MW890306 | CS-Ch-Ar | 21 | -35.856 | -71.204 |
| MW890307 | CS-Ch-Ar | 22 | -35.865 | -71.121 |
| MW890308 | CS-Ch-Ar | 22 | -35.865 | -71.121 |
| MW890309 | CS-Ch-Ar | 22 | -35.865 | -71.121 |
| MW890506 | CS-Ch-Ar | 23 | -36.164 | -72.421 |
| MW890507 | CS-Ch-Ar | 23 | -36.164 | -72.421 |
| MW890508 | CS-Ch-Ar | 23 | -36.164 | -72.421 |
| MW890509 | CS-Ch-Ar | 23 | -36.164 | -72.421 |
| MW890510 | CS-Ch-Ar | 23 | -36.164 | -72.421 |
| AY275111 | CS-Ch-Ar | 24 | -37.081 | -70.093 |
| AF297880 | CS-Ch-Ar | 25 | -37.098 | -72.551 |
| AF297881 | CS-Ch-Ar | 25 | -37.098 | -72.551 |
| MW890515 | CS-Ch-Ar | 26 | -39.399 | -71.337 |
| MW890516 | CS-Ch-Ar | 26 | -39.399 | -71.337 |
| HM167812 | CS-Ch-Ar | 27 | -39.421 | -71.391 |
| AF027309 | CS-Ch-Ar | 28 | -39.629 | -73.189 |
| AF027310 | CS-Ch-Ar | 28 | -39.629 | -73.189 |
| MW890303 | CS-Ch-Ar | 29 | -39.777 | -73.305 |
| MW890304 | CS-Ch-Ar | 29 | -39.777 | -73.305 |
| MW890321 | CS-Ch-Ar | 29 | -39.777 | -73.305 |
| MW890322 | CS-Ch-Ar | 29 | -39.777 | -73.305 |
| MW890323 | CS-Ch-Ar | 29 | -39.777 | -73.305 |
| MW890324 | CS-Ch-Ar | 29 | -39.777 | -73.305 |
| MW890325 | CS-Ch-Ar | 29 | -39.777 | -73.305 |
| MW890326 | CS-Ch-Ar | 29 | -39.777 | -73.305 |
| MW890521 | CS-Ch-Ar | 29 | -39.777 | -73.305 |
| MW890522 | CS-Ch-Ar | 29 | -39.777 | -73.305 |
| MW890523 | CS-Ch-Ar | 29 | -39.777 | -73.305 |
| MW890524 | CS-Ch-Ar | 29 | -39.777 | -73.305 |
| MW890525 | CS-Ch-Ar | 29 | -39.777 | -73.305 |
| MW890526 | CS-Ch-Ar | 29 | -39.777 | -73.305 |
| MW890298 | CS-Ch-Ar | 30 | -39.806 | -73.258 |
| MW890338 | CS-Ch-Ar | 31 | -39.941 | -73.306 |
| MW890319 | CS-Ch-Ar | 32 | -39.965 | -73.594 |
| MW890320 | CS-Ch-Ar | 32 | -39.965 | -73.594 |
| AF027324 | CS-Ch-Ar | 33 | -40.516 | -71.061 |
| AF027325 | CS-Ch-Ar | 33 | -40.516 | -71.061 |
| AF027307 | CS-Ch-Ar | 34 | -40.588 | -73.738 |
| AF027308 | CS-Ch-Ar | 34 | -40.588 | -73.738 |
| AF027311 | CS-Ch-Ar | 35 | -40.919 | -72.916 |
| AF027312 | CS-Ch-Ar | 36 | -41.093 | -71.461 |
| AF027313 | CS-Ch-Ar | 36 | -41.093 | -71.461 |
| AF027318 | CS-Ch-Ar | 37 | -41.111 | -71.181 |
| AF027319 | CS-Ch-Ar | 37 | -41.111 | -71.181 |
| AF027329 | CS-Ch-Ar | 38 | -41.102 | -70.212 |
| AF027330 | CS-Ch-Ar | 38 | -41.102 | -70.212 |
| AF027320 | CS-Ch-Ar | 39 | -41.113 | -71.414 |
| AF027321 | CS-Ch-Ar | 39 | -41.113 | -71.414 |
| AF027322 | CS-Ch-Ar | 39 | -41.113 | -71.414 |
| AF027323 | CS-Ch-Ar | 40 | -41.136 | -71.257 |
| AF027326 | CS-Ch-Ar | 41 | -41.243 | -71.147 |
| AF027327 | CS-Ch-Ar | 41 | -41.243 | -71.147 |
| AF027328 | CS-Ch-Ar | 41 | -41.243 | -71.147 |
| AF027314 | CS-Ch-Ar | 42 | -41.257 | -71.202 |
| AF027315 | CS-Ch-Ar | 42 | -41.257 | -71.202 |
| AF027316 | CS-Ch-Ar | 43 | -41.451 | -71.481 |
| AF027317 | CS-Ch-Ar | 43 | -41.451 | -71.481 |
| HM167800 | CS-Ch-Ar | 44 | -41.451 | -66.915 |
| KF234180 | CS-Ch-Ar | 44 | -41.451 | -66.915 |
| HM167799 | CS-Ch-Ar | 45 | -41.513 | -67.125 |
| MW890518 | CS-Ch-Ar | 46 | -41.535 | -70.677 |
| MW890520 | CS-Ch-Ar | 46 | -41.535 | -70.677 |
| KF234162 | CS-Ch-Ar | 47 | -41.641 | -72.211 |
| KF234174 | CS-Ch-Ar | 47 | -41.641 | -72.211 |
| MW890282 | CS-Ch-Ar | 47 | -41.641 | -72.211 |
| MW890283 | CS-Ch-Ar | 47 | -41.641 | -72.211 |
| MW890284 | CS-Ch-Ar | 47 | -41.641 | -72.211 |
| MW890285 | CS-Ch-Ar | 47 | -41.641 | -72.211 |
| MW890274 | CS-Ch-Ar | 48 | -41.646 | -72.173 |
| MW890275 | CS-Ch-Ar | 48 | -41.646 | -72.173 |
| MW890276 | CS-Ch-Ar | 48 | -41.646 | -72.173 |
| MW890277 | CS-Ch-Ar | 48 | -41.646 | -72.173 |
| MW890278 | CS-Ch-Ar | 48 | -41.646 | -72.173 |
| MW890279 | CS-Ch-Ar | 48 | -41.646 | -72.173 |
| MW890280 | CS-Ch-Ar | 48 | -41.646 | -72.173 |
| MW890281 | CS-Ch-Ar | 48 | -41.646 | -72.173 |
| MW890286 | CS-Ch-Ar | 48 | -41.646 | -72.173 |
| HM167801 | CS-Ch-Ar | 49 | -41.674 | -67.149 |
| KF234161 | CS-Ch-Ar | 50 | -41.682 | -72.328 |
| MW890287 | CS-Ch-Ar | 50 | -41.682 | -72.328 |
| MW890288 | CS-Ch-Ar | 50 | -41.682 | -72.328 |
| MW890289 | CS-Ch-Ar | 50 | -41.682 | -72.328 |
| MW890290 | CS-Ch-Ar | 50 | -41.682 | -72.328 |
| MW890291 | CS-Ch-Ar | 50 | -41.682 | -72.328 |
| MW890292 | CS-Ch-Ar | 50 | -41.682 | -72.328 |
| MW890293 | CS-Ch-Ar | 50 | -41.682 | -72.328 |
| MW890294 | CS-Ch-Ar | 50 | -41.682 | -72.328 |
| MW890299 | CS-Ch-Ar | 51 | -41.878 | -71.931 |
| MW890300 | CS-Ch-Ar | 51 | -41.878 | -71.931 |
| MW890301 | CS-Ch-Ar | 51 | -41.878 | -71.931 |
| MW890302 | CS-Ch-Ar | 51 | -41.878 | -71.931 |
| KF234177 | CS-Ch-Ar | 52 | -41.881 | -73.665 |
| KF234179 | CS-Ch-Ar | 52 | -41.881 | -73.665 |
| MW890538 | CS-Ch-Ar | 52 | -41.881 | -73.665 |
| MW890539 | CS-Ch-Ar | 52 | -41.881 | -73.665 |
| MW890485 | CS-Ch-Ar | 53 | -42.058 | -71.155 |
| MW890486 | CS-Ch-Ar | 53 | -42.058 | -71.155 |
| MW890519 | CS-Ch-Ar | 53 | -42.058 | -71.155 |
| HM167808 | CS-Ch-Ar | 54 | -42.092 | -71.625 |
| KF234164 | CS-Ch-Ar | 54 | -42.092 | -71.625 |
| KF234181 | CS-Ch-Ar | 54 | -42.092 | -71.625 |
| KF234186 | CS-Ch-Ar | 54 | -42.092 | -71.625 |
| KF234187 | CS-Ch-Ar | 54 | -42.092 | -71.625 |
| MW890421 | CS-Ch-Ar | 54 | -42.092 | -71.625 |
| MW890422 | CS-Ch-Ar | 54 | -42.092 | -71.625 |
| HM167802 | CS-Ch-Ar | 55 | -42.225 | -68.271 |
| HM167804 | CS-Ch-Ar | 55 | -42.225 | -68.271 |
| HM167805 | CS-Ch-Ar | 55 | -42.225 | -68.271 |
| HM167806 | CS-Ch-Ar | 55 | -42.225 | -68.271 |
| KF234147 | CS-Ch-Ar | 55 | -42.225 | -68.271 |
| KF234148 | CS-Ch-Ar | 55 | -42.225 | -68.271 |
| KF234191 | CS-Ch-Ar | 55 | -42.225 | -68.271 |
| KJ614633 | CS-Ch-Ar | 55 | -42.225 | -68.271 |
| MW890410 | CS-Ch-Ar | 55 | -42.225 | -68.271 |
| MW890411 | CS-Ch-Ar | 55 | -42.225 | -68.271 |
| MW890504 | CS-Ch-Ar | 55 | -42.225 | -68.271 |
| MW890484 | CS-Ch-Ar | 56 | -42.331 | -70.551 |
| KF234170 | CS-Ch-Ar | 57 | -42.463 | -73.808 |
| HM167807 | CS-Ch-Ar | 58 | -42.668 | -70.093 |
| MW890412 | CS-Ch-Ar | 58 | -42.668 | -70.093 |
| MW890413 | CS-Ch-Ar | 58 | -42.668 | -70.093 |
| MW890414 | CS-Ch-Ar | 58 | -42.668 | -70.093 |
| MW890415 | CS-Ch-Ar | 58 | -42.668 | -70.093 |
| MW890416 | CS-Ch-Ar | 58 | -42.668 | -70.093 |
| MW890417 | CS-Ch-Ar | 58 | -42.668 | -70.093 |
| MW890418 | CS-Ch-Ar | 58 | -42.668 | -70.093 |
| MW890419 | CS-Ch-Ar | 58 | -42.668 | -70.093 |
| MW890420 | CS-Ch-Ar | 58 | -42.668 | -70.093 |
| KF234182 | CS-Ch-Ar | 59 | -42.891 | -71.567 |
| KF234183 | CS-Ch-Ar | 59 | -42.891 | -71.567 |
| KF234184 | CS-Ch-Ar | 59 | -42.891 | -71.567 |
| KF234185 | CS-Ch-Ar | 59 | -42.891 | -71.567 |
| MW890423 | CS-Ch-Ar | 59 | -42.891 | -71.567 |
| MW890424 | CS-Ch-Ar | 59 | -42.891 | -71.567 |
| MW890425 | CS-Ch-Ar | 59 | -42.891 | -71.567 |
| MW890426 | CS-Ch-Ar | 59 | -42.891 | -71.567 |
| MW890427 | CS-Ch-Ar | 59 | -42.891 | -71.567 |
| KF234178 | CS-Ch-Ar | 60 | -42.898 | -71.597 |
| MW890517 | CS-Ch-Ar | 61 | -42.908 | -71.611 |
| MW890362 | CS-Ch-Ar | 62 | -43.104 | -73.928 |
| MW890363 | CS-Ch-Ar | 62 | -43.104 | -73.928 |
| MW890364 | CS-Ch-Ar | 62 | -43.104 | -73.928 |
| MW890357 | CS-Ch-Ar | 63 | -43.114 | -73.943 |
| MW890358 | CS-Ch-Ar | 63 | -43.114 | -73.943 |
| MW890359 | CS-Ch-Ar | 63 | -43.114 | -73.943 |
| MW890360 | CS-Ch-Ar | 63 | -43.114 | -73.943 |
| MW890361 | CS-Ch-Ar | 63 | -43.114 | -73.943 |
| KF234167 | CS-Ch-Ar | 64 | -43.121 | -73.944 |
| MW890271 | CS-Ch-Ar | 64 | -43.121 | -73.944 |
| MW890272 | CS-Ch-Ar | 64 | -43.121 | -73.944 |
| MW890273 | CS-Ch-Ar | 64 | -43.121 | -73.944 |
| MW890428 | CS-Ch-Ar | 65 | -43.711 | -70.352 |
| KF234163 | CS-Ch-Ar | 66 | -43.871 | -70.731 |
| KF234163 | CS-Ch-Ar | 66 | -43.871 | -70.731 |
| KF234165 | CS-Ch-Ar | 66 | -43.871 | -70.731 |
| KF234171 | CS-Ch-Ar | 66 | -43.871 | -70.731 |
| KF234172 | CS-Ch-Ar | 66 | -43.871 | -70.731 |
| KF234175 | CS-Ch-Ar | 66 | -43.871 | -70.731 |
| KF234176 | CS-Ch-Ar | 66 | -43.871 | -70.731 |
| KF234158 | CS-Ch-Ar | 67 | -44.111 | -67.991 |
| MW890429 | CS-Ch-Ar | 67 | -44.111 | -67.991 |
| MW890430 | CS-Ch-Ar | 67 | -44.111 | -67.991 |
| MW890431 | CS-Ch-Ar | 67 | -44.111 | -67.991 |
| MW890432 | CS-Ch-Ar | 67 | -44.111 | -67.991 |
| AF297884 | CS-Ch-Ar | 68 | -45.461 | -71.991 |
| AF297885 | CS-Ch-Ar | 68 | -45.461 | -71.991 |
| AF297889 | CS-Ch-Ar | 69 | -45.481 | -71.539 |
| AF297890 | CS-Ch-Ar | 69 | -45.481 | -71.539 |
| AF297882 | CS-Ch-Ar | 70 | -45.485 | -72.658 |
| AF297883 | CS-Ch-Ar | 70 | -45.485 | -72.658 |
| AF297886 | CS-Ch-Ar | 71 | -45.485 | -71.627 |
| AF297887 | CS-Ch-Ar | 71 | -45.485 | -71.627 |
| AF297888 | CS-Ch-Ar | 71 | -45.485 | -71.627 |
| KF234154 | CS-Ch-Ar | 72 | -45.515 | -67.494 |
| MW890495 | CS-Ch-Ar | 72 | -45.515 | -67.494 |
| MW890496 | CS-Ch-Ar | 72 | -45.515 | -67.494 |
| MW890497 | CS-Ch-Ar | 72 | -45.515 | -67.494 |
| MW890498 | CS-Ch-Ar | 72 | -45.515 | -67.494 |
| MW890499 | CS-Ch-Ar | 72 | -45.515 | -67.494 |
| MW890500 | CS-Ch-Ar | 72 | -45.515 | -67.494 |
| MW890501 | CS-Ch-Ar | 72 | -45.515 | -67.494 |
| MW890502 | CS-Ch-Ar | 72 | -45.515 | -67.494 |
| MW890503 | CS-Ch-Ar | 72 | -45.515 | -67.494 |
| AF297896 | CS-Ch-Ar | 73 | -45.573 | -72.086 |
| AF297891 | CS-Ch-Ar | 74 | -45.908 | -71.658 |
| AF297892 | CS-Ch-Ar | 74 | -45.908 | -71.658 |
| AF297893 | CS-Ch-Ar | 74 | -45.908 | -71.658 |
| AF297898 | CS-Ch-Ar | 74 | -45.908 | -71.658 |
| AF297899 | CS-Ch-Ar | 74 | -45.908 | -71.658 |
| AF297900 | CS-Ch-Ar | 74 | -45.908 | -71.658 |
| AF297897 | CS-Ch-Ar | 75 | -45.921 | -71.694 |
| KF234173 | CS-Ch-Ar | 76 | -45.951 | -71.531 |
| AF297894 | CS-Ch-Ar | 77 | -46.272 | -71.962 |
| AF297895 | CS-Ch-Ar | 77 | -46.272 | -71.962 |
| AF297902 | CS-Ch-Ar | 78 | -46.543 | -71.716 |
| AF297901 | CS-Ch-Ar | 79 | -46.548 | -71.729 |
| KJ614631 | CS-Ch-Ar | 80 | -47.777 | -73.304 |
| HM167811 | CS-Ch-Ar | 81 | -47.878 | -66.426 |
| MW890482 | CS-Ch-Ar | 81 | -47.878 | -66.426 |
| MW890483 | CS-Ch-Ar | 81 | -47.878 | -66.426 |
| MW890487 | CS-Ch-Ar | 81 | -47.878 | -66.426 |
| MW890488 | CS-Ch-Ar | 81 | -47.878 | -66.426 |
| MW890489 | CS-Ch-Ar | 81 | -47.878 | -66.426 |
| MW890490 | CS-Ch-Ar | 81 | -47.878 | -66.426 |
| MW890491 | CS-Ch-Ar | 81 | -47.878 | -66.426 |
| MW890492 | CS-Ch-Ar | 81 | -47.878 | -66.426 |
| MW890493 | CS-Ch-Ar | 81 | -47.878 | -66.426 |
| MW890494 | CS-Ch-Ar | 81 | -47.878 | -66.426 |
| HM167810 | CS-Ch-Ar | 82 | -48.996 | -70.259 |
| KF234150 | CS-Ch-Ar | 82 | -48.996 | -70.259 |
| KF234159 | CS-Ch-Ar | 82 | -48.996 | -70.259 |
| MW890476 | CS-Ch-Ar | 82 | -48.996 | -70.259 |
| MW890477 | CS-Ch-Ar | 82 | -48.996 | -70.259 |
| MW890478 | CS-Ch-Ar | 82 | -48.996 | -70.259 |
| MW890479 | CS-Ch-Ar | 82 | -48.996 | -70.259 |
| MW890480 | CS-Ch-Ar | 82 | -48.996 | -70.259 |
| MW890481 | CS-Ch-Ar | 82 | -48.996 | -70.259 |
| EF118755 | CS-Ch-Ar | 83 | -49.104 | -74.275 |
| EF118754 | CS-Ch-Ar | 84 | -49.292 | -74.742 |
| EF118756 | CS-Ch-Ar | 84 | -49.292 | -74.742 |
| KF234151 | CS-Ch-Ar | 85 | -50.117 | -68.414 |
| KF234152 | CS-Ch-Ar | 85 | -50.117 | -68.414 |
| KF234153 | CS-Ch-Ar | 85 | -50.117 | -68.414 |
| HM167813 | CS-Ch-Ar | 86 | -51.132 | -72.826 |
| KF234160 | CS-Ch-Ar | 86 | -51.132 | -72.826 |
| MW890527 | CS-Ch-Ar | 86 | -51.132 | -72.826 |
| MW890528 | CS-Ch-Ar | 86 | -51.132 | -72.826 |
| MW890529 | CS-Ch-Ar | 86 | -51.132 | -72.826 |
| MW890530 | CS-Ch-Ar | 86 | -51.132 | -72.826 |
| MW890531 | CS-Ch-Ar | 86 | -51.132 | -72.826 |
| MW890532 | CS-Ch-Ar | 86 | -51.132 | -72.826 |
| MW890533 | CS-Ch-Ar | 86 | -51.132 | -72.826 |
| MW890534 | CS-Ch-Ar | 86 | -51.132 | -72.826 |
| MW890535 | CS-Ch-Ar | 86 | -51.132 | -72.826 |
| MW890536 | CS-Ch-Ar | 86 | -51.132 | -72.826 |
| MW890537 | CS-Ch-Ar | 86 | -51.132 | -72.826 |
| MW890375 | CS-Ch-Ar | 89 | -53.151 | -70.961 |
| MW890377 | CS-Ch-Ar | 89 | -53.151 | -70.961 |
| MW890378 | CS-Ch-Ar | 89 | -53.151 | -70.961 |
| MW890379 | CS-Ch-Ar | 89 | -53.151 | -70.961 |
| MW890380 | CS-Ch-Ar | 89 | -53.151 | -70.961 |
| MW890383 | CS-Ch-Ar | 89 | -53.151 | -70.961 |
| MW890384 | CS-Ch-Ar | 89 | -53.151 | -70.961 |
| MW890385 | CS-Ch-Ar | 90 | -53.606 | -70.938 |
| MW890386 | CS-Ch-Ar | 90 | -53.606 | -70.938 |
| MW890387 | CS-Ch-Ar | 90 | -53.606 | -70.938 |
| MW890388 | CS-Ch-Ar | 90 | -53.606 | -70.938 |
| MW890390 | CS-Ch-Ar | 90 | -53.606 | -70.938 |
| MW890391 | CS-Ch-Ar | 90 | -53.606 | -70.938 |
| KJ614630 | Men-Ar | 10 | -32.932 | -68.921 |
| KP665997 | Men-Ar | 16 | -35.141 | -70.195 |
| AY341034 | N-Ch | 1 | -19.194 | -70.262 |
| AY750846 | N-Ch | 1 | -19.194 | -70.262 |
| AY341035 | N-Ch | 2 | -19.985 | -69.595 |
| AY750847 | N-Ch | 2 | -19.985 | -69.595 |
| AF027305 | N-Ch | 3 | -29.938 | -71.167 |
| AF297878 | N-Ch | 3 | -29.938 | -71.167 |
| MW890339 | N-Ch | 4 | -30.271 | -71.484 |
| MW890340 | N-Ch | 4 | -30.271 | -71.484 |
| MW890341 | N-Ch | 4 | -30.271 | -71.484 |
| MW890342 | N-Ch | 4 | -30.271 | -71.484 |
| MW890343 | N-Ch | 5 | -30.281 | -71.461 |
| MW890344 | N-Ch | 5 | -30.281 | -71.461 |
| MW890345 | N-Ch | 5 | -30.281 | -71.461 |
| MW890346 | N-Ch | 5 | -30.281 | -71.461 |
| MW890347 | N-Ch | 5 | -30.281 | -71.461 |
| MW890348 | N-Ch | 5 | -30.281 | -71.461 |
| MW890349 | N-Ch | 5 | -30.281 | -71.461 |
| MW890350 | N-Ch | 5 | -30.281 | -71.461 |
| MW890511 | N-Ch | 6 | -30.734 | -71.686 |
| MW890512 | N-Ch | 6 | -30.734 | -71.686 |
| MW890264 | N-Ch | 7 | -31.782 | -70.972 |
| MW890265 | N-Ch | 7 | -31.782 | -70.972 |
| MW890266 | N-Ch | 7 | -31.782 | -70.972 |
| MW890267 | N-Ch | 7 | -31.782 | -70.972 |
| MW890513 | N-Ch | 8 | -31.867 | -71.153 |
| MW890514 | N-Ch | 8 | -31.867 | -71.153 |
| MW890409 | N-Ch | 9 | -32.161 | -71.492 |
| MW890505 | N-Ch | 11 | -33.171 | -71.457 |
| AF027306 | N-Ch | 12 | -33.371 | -71.665 |
| AF297879 | N-Ch | 12 | -33.371 | -71.665 |
| MW890295 | N-Ch | 13 | -33.833 | -70.066 |
| MW890296 | N-Ch | 13 | -33.833 | -70.066 |
| MW890297 | N-Ch | 13 | -33.833 | -70.066 |
| MW890351 | N-Ch | 14 | -34.461 | -72.024 |
| MW890352 | N-Ch | 14 | -34.461 | -72.024 |
| MW890353 | N-Ch | 14 | -34.461 | -72.024 |
| MW890354 | N-Ch | 14 | -34.461 | -72.024 |
| MW890355 | N-Ch | 14 | -34.461 | -72.024 |
| MW890356 | N-Ch | 14 | -34.461 | -72.024 |
| MW890311 | N-Ch | 17 | -35.161 | -72.254 |
| MW890312 | N-Ch | 17 | -35.161 | -72.254 |
| MW890317 | N-Ch | 17 | -35.161 | -72.254 |
| MW890318 | N-Ch | 17 | -35.161 | -72.254 |
| EU683435 | TdF-SCh | 87 | -52.73 | -68.559 |
| KF234204 | TdF-SCh | 87 | -52.731 | -68.559 |
| KJ614634 | TdF-SCh | 87 | -52.731 | -68.559 |
| MW890472 | TdF-SCh | 87 | -52.731 | -68.559 |
| MW890473 | TdF-SCh | 87 | -52.731 | -68.559 |
| MW890474 | TdF-SCh | 87 | -52.731 | -68.559 |
| MW890268 | TdF-SCh | 88 | -52.849 | -71.951 |
| MW890269 | TdF-SCh | 88 | -52.849 | -71.951 |
| MW890270 | TdF-SCh | 88 | -52.849 | -71.951 |
| MW890376 | TdF-SCh | 89 | -53.151 | -70.961 |
| MW890381 | TdF-SCh | 89 | -53.151 | -70.961 |
| MW890382 | TdF-SCh | 89 | -53.151 | -70.961 |
| MW890389 | TdF-SCh | 90 | -53.606 | -70.938 |
| KF234193 | TdF-SCh | 91 | -53.669 | -68.467 |
| KF234198 | TdF-SCh | 91 | -53.669 | -68.467 |
| KF234199 | TdF-SCh | 91 | -53.669 | -68.467 |
| KF234199 | TdF-SCh | 91 | -53.669 | -68.467 |
| KF234201 | TdF-SCh | 91 | -53.669 | -68.467 |
| KF234202 | TdF-SCh | 91 | -53.669 | -68.467 |
| KF234205 | TdF-SCh | 92 | -53.788 | -67.676 |
| MW890475 | TdF-SCh | 92 | -53.788 | -67.676 |
| HM167792 | TdF-SCh | 93 | -53.825 | -67.795 |
| KF234194 | TdF-SCh | 93 | -53.825 | -67.795 |
| KF234195 | TdF-SCh | 93 | -53.825 | -67.795 |
| KF234206 | TdF-SCh | 93 | -53.825 | -67.795 |
| MW890433 | TdF-SCh | 93 | -53.825 | -67.795 |
| MW890434 | TdF-SCh | 93 | -53.825 | -67.795 |
| MW890435 | TdF-SCh | 93 | -53.825 | -67.795 |
| MW890436 | TdF-SCh | 93 | -53.825 | -67.795 |
| MW890437 | TdF-SCh | 93 | -53.825 | -67.795 |
| MW890438 | TdF-SCh | 93 | -53.825 | -67.795 |
| MW890439 | TdF-SCh | 93 | -53.825 | -67.795 |
| MW890404 | TdF-SCh | 94 | -54.142 | -68.726 |
| MW890405 | TdF-SCh | 94 | -54.142 | -68.726 |
| MW890406 | TdF-SCh | 94 | -54.142 | -68.726 |
| MW890399 | TdF-SCh | 94 | -54.142 | -68.726 |
| MW890403 | TdF-SCh | 94 | -54.142 | -68.726 |
| MW890407 | TdF-SCh | 94 | -54.142 | -68.726 |
| MW890408 | TdF-SCh | 94 | -54.142 | -68.726 |
| MW890400 | TdF-SCh | 94 | -54.142 | -68.726 |
| MW890401 | TdF-SCh | 94 | -54.142 | -68.726 |
| MW890402 | TdF-SCh | 94 | -54.142 | -68.726 |
| EU840992 | TdF-SCh | 95 | -54.181 | -71.368 |
| EU840993 | TdF-SCh | 95 | -54.181 | -71.368 |
| HM167793 | TdF-SCh | 96 | -54.484 | -66.432 |
| MW890440 | TdF-SCh | 96 | -54.484 | -66.432 |
| MW890441 | TdF-SCh | 96 | -54.484 | -66.432 |
| MW890442 | TdF-SCh | 96 | -54.484 | -66.432 |
| MW890443 | TdF-SCh | 96 | -54.484 | -66.432 |
| MW890444 | TdF-SCh | 96 | -54.484 | -66.432 |
| MW890445 | TdF-SCh | 96 | -54.484 | -66.432 |
| MW890446 | TdF-SCh | 96 | -54.484 | -66.432 |
| MW890447 | TdF-SCh | 96 | -54.484 | -66.432 |
| HM167794 | TdF-SCh | 97 | -54.611 | -67.643 |
| MW890448 | TdF-SCh | 97 | -54.611 | -67.643 |
| MW890449 | TdF-SCh | 97 | -54.611 | -67.643 |
| MW890450 | TdF-SCh | 97 | -54.611 | -67.643 |
| HM167795 | TdF-SCh | 98 | -54.621 | -67.429 |
| HM167797 | TdF-SCh | 99 | -54.789 | -68.394 |
| KF234191 | TdF-SCh | 99 | -54.789 | -68.394 |
| KF234196 | TdF-SCh | 99 | -54.789 | -68.394 |
| KF234197 | TdF-SCh | 99 | -54.789 | -68.394 |
| MW890455 | TdF-SCh | 99 | -54.789 | -68.394 |
| MW890456 | TdF-SCh | 99 | -54.789 | -68.394 |
| MW890365 | TdF-SCh | 100 | -54.822 | -68.324 |
| MW890366 | TdF-SCh | 100 | -54.822 | -68.324 |
| MW890367 | TdF-SCh | 100 | -54.822 | -68.324 |
| MW890368 | TdF-SCh | 100 | -54.822 | -68.324 |
| MW890369 | TdF-SCh | 100 | -54.822 | -68.324 |
| MW890370 | TdF-SCh | 100 | -54.822 | -68.324 |
| MW890371 | TdF-SCh | 100 | -54.822 | -68.324 |
| MW890372 | TdF-SCh | 100 | -54.822 | -68.324 |
| MW890373 | TdF-SCh | 100 | -54.822 | -68.324 |
| MW890374 | TdF-SCh | 100 | -54.822 | -68.324 |
| HM167796 | TdF-SCh | 101 | -54.827 | -68.315 |
| KF234192 | TdF-SCh | 101 | -54.827 | -68.315 |
| KF234194 | TdF-SCh | 101 | -54.827 | -68.315 |
| MW890451 | TdF-SCh | 101 | -54.827 | -68.315 |
| MW890452 | TdF-SCh | 101 | -54.827 | -68.315 |
| MW890453 | TdF-SCh | 101 | -54.827 | -68.315 |
| MW890454 | TdF-SCh | 101 | -54.827 | -68.315 |
| MW890457 | TdF-SCh | 101 | -54.827 | -68.315 |
| MW890458 | TdF-SCh | 101 | -54.827 | -68.315 |
| MW890460 | TdF-SCh | 101 | -54.827 | -68.315 |
| MW890461 | TdF-SCh | 101 | -54.827 | -68.315 |
| MW890462 | TdF-SCh | 101 | -54.827 | -68.315 |
| KF234202 | TdF-SCh | 102 | -54.847 | -68.482 |
| HM167798 | TdF-SCh | 103 | -54.872 | -67.423 |
| MW890459 | TdF-SCh | 103 | -54.872 | -67.423 |
| MW890463 | TdF-SCh | 103 | -54.872 | -67.423 |
| MW890464 | TdF-SCh | 103 | -54.872 | -67.423 |
| MW890465 | TdF-SCh | 103 | -54.872 | -67.423 |
| MW890466 | TdF-SCh | 103 | -54.872 | -67.423 |
| MW890467 | TdF-SCh | 103 | -54.872 | -67.423 |
| MW890468 | TdF-SCh | 103 | -54.872 | -67.423 |
| MW890469 | TdF-SCh | 103 | -54.872 | -67.423 |
| MW890470 | TdF-SCh | 103 | -54.872 | -67.423 |
| MW890471 | TdF-SCh | 103 | -54.872 | -67.423 |

**Table S1.** Correlation matrix based on Pearson's correlation constructed using the characterization of the climatic attributes of the geographic distribution of *Abrothrix olivacea*. Bioclimatic variables whose values of correlation are less than 0.75 or superior to -0.75 (regarded here as uncorrelated) are indicated in bold.

|  | Bio_01 | Bio_02 | **Bio_03** | **Bio_04** | Bio_05 | **Bio_06** | Bio_07 | Bio_08 | **Bio_09** | Bio_10 | Bio_11 | Bio_12 | Bio_13 | Bio_14 | **Bio_15** | **Bio_16** | Bio_17 | Bio_18 | Bio_19 |
| --- | --- | --- | --- | --- | --- | --- | --- | --- | --- | --- | --- | --- | --- | --- | --- | --- | --- | --- | --- |
| Bio_01 | 1 | 0.3655 | -0.1326 | 0.4586 | 0.9126 | 0.7805 | 0.5059 | 0.8093 | 0.5806 | 0.9681 | 0.9461 | -0.2182 | -0.1704 | -0.2654 | 0.169 | -0.1678 | -0.2622 | -0.1734 | -0.2118 |
| Bio_02 | 0.3655 | 1 | 0.444 | 0.5297 | 0.6108 | -0.232 | 0.8738 | 0.4286 | 0.0308 | 0.4463 | 0.2037 | -0.5804 | -0.4533 | -0.6436 | 0.5205 | -0.4689 | -0.6352 | -0.4983 | -0.5259 |
| **Bio_03** | -0.1326 | 0.444 | 1 | -0.5076 | -0.1717 | -0.2232 | -0.041 | 0.0183 | -0.1719 | -0.2612 | 0.0259 | -0.2563 | -0.1676 | -0.2924 | 0.7173 | -0.1831 | -0.288 | -0.2358 | -0.196 |
| **Bio_04** | 0.4586 | 0.5297 | -0.5076 | 1 | 0.7387 | -0.0189 | 0.8711 | 0.3483 | 0.2244 | 0.6642 | 0.1485 | -0.385 | -0.3551 | -0.4002 | -0.2228 | -0.3539 | -0.3953 | -0.3323 | -0.3794 |
| Bio_05 | 0.9126 | 0.6108 | -0.1717 | 0.7387 | 1 | 0.5169 | 0.7944 | 0.7233 | 0.5274 | 0.9743 | 0.7489 | -0.3532 | -0.2666 | -0.4286 | 0.1368 | -0.2678 | -0.4217 | -0.3271 | -0.3094 |
| **Bio_06** | 0.7805 | -0.232 | -0.2232 | -0.0189 | 0.5169 | 1 | -0.1092 | 0.5011 | 0.6463 | 0.6598 | 0.8856 | 0.1788 | 0.1884 | 0.1265 | -0.0356 | 0.1986 | 0.1272 | 0.1095 | 0.2057 |
| Bio_07 | 0.5059 | 0.8738 | -0.041 | 0.8711 | 0.7944 | -0.1092 | 1 | 0.4843 | 0.1539 | 0.6633 | 0.2412 | -0.537 | -0.4433 | -0.5874 | 0.1841 | -0.4518 | -0.58 | -0.4576 | -0.5052 |
| Bio_08 | 0.8093 | 0.4286 | 0.0183 | 0.3483 | 0.7233 | 0.5011 | 0.4843 | 1 | 0.0786 | 0.7688 | 0.7643 | -0.1975 | -0.2021 | -0.1874 | 0.2223 | -0.2021 | -0.1875 | -0.015 | -0.3064 |
| **Bio_09** | 0.5806 | 0.0308 | -0.1719 | 0.2244 | 0.5274 | 0.6463 | 0.1539 | 0.0786 | 1 | 0.562 | 0.5766 | -0.1232 | -0.0143 | -0.2367 | 0.0605 | -0.011 | -0.2301 | -0.3129 | 0.0579 |
| Bio_10 | 0.9681 | 0.4463 | -0.2612 | 0.6642 | 0.9743 | 0.6598 | 0.6633 | 0.7688 | 0.562 | 1 | 0.8377 | -0.278 | -0.2272 | -0.3263 | 0.0683 | -0.2245 | -0.3221 | -0.2342 | -0.2663 |
| Bio_11 | 0.9461 | 0.2037 | 0.0259 | 0.1485 | 0.7489 | 0.8856 | 0.2412 | 0.7643 | 0.5766 | 0.8377 | 1 | -0.0902 | -0.0458 | -0.1416 | 0.2534 | -0.0431 | -0.1396 | -0.0691 | -0.0797 |
| Bio_12 | -0.2182 | -0.5804 | -0.2563 | -0.385 | -0.3532 | 0.1788 | -0.537 | -0.1975 | -0.1232 | -0.278 | -0.0902 | 1 | 0.9423 | 0.9353 | -0.2588 | 0.9499 | 0.9445 | 0.9034 | 0.9237 |
| Bio_13 | -0.1704 | -0.4533 | -0.1676 | -0.3551 | -0.2666 | 0.1884 | -0.4433 | -0.2021 | -0.0143 | -0.2272 | -0.0458 | 0.9423 | 1 | 0.7715 | -0.0876 | 0.9981 | 0.7849 | 0.7496 | 0.9771 |
| Bio_14 | -0.2654 | -0.6436 | -0.2924 | -0.4002 | -0.4286 | 0.1265 | -0.5874 | -0.1874 | -0.2367 | -0.3263 | -0.1416 | 0.9353 | 0.7715 | 1 | -0.3708 | 0.7833 | 0.9976 | 0.953 | 0.7542 |
| **Bio_15** | 0.169 | 0.5205 | 0.7173 | -0.2228 | 0.1368 | -0.0356 | 0.1841 | 0.2223 | 0.0605 | 0.0683 | 0.2534 | -0.2588 | -0.0876 | -0.3708 | 1 | -0.1121 | -0.3644 | -0.254 | -0.1623 |
| **Bio_16** | -0.1678 | -0.4689 | -0.1831 | -0.3539 | -0.2678 | 0.1986 | -0.4518 | -0.2021 | -0.011 | -0.2245 | -0.0431 | 0.9499 | 0.9981 | 0.7833 | -0.1121 | 1 | 0.7966 | 0.7578 | 0.9808 |
| Bio_17 | -0.2622 | -0.6352 | -0.288 | -0.3953 | -0.4217 | 0.1272 | -0.58 | -0.1875 | -0.2301 | -0.3221 | -0.1396 | 0.9445 | 0.7849 | 0.9976 | -0.3644 | 0.7966 | 1 | 0.9587 | 0.7659 |
| Bio_18 | -0.1734 | -0.4983 | -0.2358 | -0.3323 | -0.3271 | 0.1095 | -0.4576 | -0.015 | -0.3129 | -0.2342 | -0.0691 | 0.9034 | 0.7496 | 0.953 | -0.254 | 0.7578 | 0.9587 | 1 | 0.675 |
| Bio_19 | -0.2118 | -0.5259 | -0.196 | -0.3794 | -0.3094 | 0.2057 | -0.5052 | -0.3064 | 0.0579 | -0.2663 | -0.0797 | 0.9237 | 0.9771 | 0.7542 | -0.1623 | 0.9808 | 0.7659 | 0.675 | 1 |

**Table S2.** Results of the Principal Component Analysis (PCA) performed to compare the climatic niche of the mitochondrial phylogroups of *Abrothrix olivacea*. Contributions of the bioclimatic variables to each principal component (PC) are shown together with the eigenvalue and explained variance by the first three components obtained.

| Bioclimatic variables | PC 1 | PC 2 | PC 3 |
| --- | --- | --- | --- |
| Bio 01: Annual Mean Temperature | 6.167 | 7.659 | 0.065 |
| Bio 02: Mean Diurnal Range (Mean of monthly (max temp - min temp)) | 6.834 | 0.417 | 11.034 |
| Bio 03: Isothermality (BIO2/BIO7) (×100) | 4.311 | 4.862 | 1.968 |
| Bio 04: Temperature Seasonality (standard deviation ×100) | 1.282 | 5.721 | 16.309 |
| Bio 05: Max Temperature of Warmest Month | 9.427 | 1.878 | 4.188 |
| Bio 06: Min Temperature of Coldest Month | 1.009 | 10.897 | 7.134 |
| Bio 07: Temperature Annual Range (BIO5-BIO6) | 3.410 | 3.004 | 17.854 |
| Bio 08: Mean Temperature of Wettest Quarter | 1.743 | 5.074 | 7.186 |
| Bio 09: Mean Temperature of Driest Quarter | 5.773 | 4.430 | 4.005 |
| Bio 10: Mean Temperature of Warmest Quarter | 8.279 | 4.128 | 1.389 |
| Bio 11: Mean Temperature of Coldest Quarter | 3.550 | 10.341 | 1.863 |
| Bio 12: Annual Precipitation | 6.396 | 6.235 | 3.807 |
| Bio 13: Precipitation of Wettest Month | 2.862 | 8.061 | 6.885 |
| Bio 14: Precipitation of Driest Month | 8.988 | 2.814 | 0.724 |
| Bio 15: Precipitation Seasonality (Coefficient of Variation) | 6.131 | 3.063 | 0.983 |
| Bio 16: Precipitation of Wettest Quarter | 3.230 | 8.035 | 6.483 |
| Bio 17: Precipitation of Driest Quarter | 8.856 | 2.925 | 0.957 |
| Bio 18: Precipitation of Warmest Quarter | 8.985 | 2.327 | 0.411 |
| Bio 19: Precipitation of Coldest Quarter | 2.766 | 8.128 | 6.755 |
| Eigenvalue | 7.619 | 6.447 | 2.839 |
| % of explained variance | 40.098 | 33.930 | 14.943 |

**Table S3.** Results of the calibrations performed with ENMeval R package to establish the optimal MaxEnt setup employed for constructing the ecological niche models of *Abrothrix olivacea* and three of their mitochondrial phylogroups (N-Ch, CS-Ch-Ar, TdF-SCh). For each modelling instance all combination of feature classes (fc) and regularization multipliers (rm) values employed in each model run are show. In addition, averages (avg) and standard deviation (sd) of AUC test values and the omission rate of the 10th percentile (OR 10), as well as the corrected Akaike information criterion (AICc) and its delta are shown. The bold line corresponds to the model with the lowest value of AICc, which in turns was selected as the ‘best’ setting among all configurations evaluated.

| Modelling instance | Model | fc | rm | avg AUC | sd AUC | avg OR 10 pct | sd OR 10 pct | AICc | delta AICc |
| --- | --- | --- | --- | --- | --- | --- | --- | --- | --- |
| *Abrothrix olivacea* | 1 | L | 0.5 | 0.862 | 0.097 | 0.127 | 0.184 | 9731.442 | 157.849 |
|  | 2 | L | 1 | 0.854 | 0.093 | 0.173 | 0.299 | 9574.364 | 0.771 |
|  | **3** | **L** | **1.5** | **0.872** | **0.091** | **0.111** | **0.309** | **9573.593** | **0.000** |
|  | 4 | L | 2 | 0.852 | 0.108 | 0.144 | 0.208 | 9768.065 | 194.472 |
|  | 5 | L | 2.5 | 0.850 | 0.110 | 0.141 | 0.209 | 9772.437 | 198.844 |
|  | 6 | L | 3 | 0.850 | 0.112 | 0.141 | 0.209 | 9776.255 | 202.662 |
|  | 7 | L | 3.5 | 0.849 | 0.115 | 0.141 | 0.209 | 9777.147 | 203.554 |
|  | 8 | L | 4 | 0.848 | 0.117 | 0.138 | 0.216 | 9779.831 | 206.238 |
|  | 9 | Q | 0.5 | 0.846 | 0.093 | 0.155 | 0.228 | 9769.238 | 195.645 |
|  | 10 | Q | 1 | 0.849 | 0.094 | 0.132 | 0.197 | 9773.488 | 199.895 |
|  | 11 | Q | 1.5 | 0.850 | 0.099 | 0.132 | 0.197 | 9780.883 | 207.290 |
|  | 12 | Q | 2 | 0.850 | 0.101 | 0.130 | 0.207 | 9789.244 | 215.651 |
|  | 13 | Q | 2.5 | 0.850 | 0.103 | 0.132 | 0.224 | 9792.447 | 218.854 |
|  | 14 | Q | 3 | 0.850 | 0.106 | 0.132 | 0.227 | 9798.111 | 224.518 |
|  | 15 | Q | 3.5 | 0.850 | 0.107 | 0.135 | 0.225 | 9806.595 | 233.002 |
|  | 16 | Q | 4 | 0.850 | 0.109 | 0.138 | 0.231 | 9812.439 | 238.846 |
|  | 17 | H | 0.5 | 0.858 | 0.091 | 0.228 | 0.287 | 9658.036 | 84.443 |
|  | 18 | H | 1 | 0.851 | 0.095 | 0.175 | 0.298 | 9622.668 | 49.075 |
|  | 19 | H | 1.5 | 0.855 | 0.102 | 0.132 | 0.192 | 9741.928 | 168.335 |
|  | 20 | H | 2 | 0.853 | 0.106 | 0.135 | 0.191 | 9750.899 | 177.306 |
|  | 21 | H | 2.5 | 0.871 | 0.094 | 0.178 | 0.304 | 9598.595 | 25.002 |
|  | 22 | H | 3 | 0.868 | 0.097 | 0.196 | 0.298 | 9586.901 | 13.308 |
|  | 23 | H | 3.5 | 0.862 | 0.101 | 0.202 | 0.296 | 9609.795 | 36.202 |
|  | 24 | H | 4 | 0.857 | 0.103 | 0.207 | 0.296 | 9618.197 | 44.604 |
| N-Ch | 1 | L | 0.5 | 0.764 | 0.178 | 0.142 | 0.438 | 1577.330 | 13.737 |
|  | **2** | **L** | **1** | **0.867** | **0.176** | **0.111** | **0.375** | **1563.593** | **0.000** |
|  | 3 | L | 1.5 | 0.845 | 0.164 | 0.095 | 0.344 | 1564.850 | 1.258 |
|  | 4 | L | 2 | 0.751 | 0.178 | 0.137 | 0.375 | 1581.645 | 18.053 |
|  | 5 | L | 2.5 | 0.752 | 0.176 | 0.111 | 0.375 | 1580.561 | 16.968 |
|  | 6 | L | 3 | 0.750 | 0.174 | 0.141 | 0.375 | 1581.722 | 18.129 |
|  | 7 | L | 3.5 | 0.753 | 0.168 | 0.126 | 0.406 | 1582.924 | 19.331 |
|  | 8 | L | 4 | 0.778 | 0.157 | 0.126 | 0.406 | 1584.242 | 20.649 |
|  | 9 | LQ | 0.5 | 0.817 | 0.127 | 0.111 | 0.375 | 1572.080 | 8.487 |
|  | 10 | LQ | 1 | 0.752 | 0.184 | 0.111 | 0.375 | 1577.082 | 13.489 |
|  | 11 | LQ | 1.5 | 0.713 | 0.167 | 0.095 | 0.344 | 1566.371 | 2.778 |
|  | 12 | LQ | 2 | 0.751 | 0.180 | 0.111 | 0.375 | 1578.907 | 15.314 |
|  | 13 | LQ | 2.5 | 0.763 | 0.173 | 0.192 | 0.375 | 1571.104 | 7.511 |
|  | 14 | LQ | 3 | 0.758 | 0.162 | 0.111 | 0.375 | 1572.717 | 9.124 |
|  | 15 | LQ | 3.5 | 0.760 | 0.152 | 0.163 | 0.375 | 1577.320 | 13.727 |
|  | 16 | LQ | 4 | 0.765 | 0.142 | 0.111 | 0.375 | 1582.696 | 19.103 |
|  | 17 | Q | 0.5 | 0.725 | 0.171 | 0.173 | 0.385 | 1593.264 | 29.672 |
|  | 18 | Q | 1 | 0.725 | 0.168 | 0.157 | 0.355 | 1594.905 | 31.312 |
|  | 19 | Q | 1.5 | 0.728 | 0.166 | 0.171 | 0.375 | 1597.556 | 33.963 |
|  | 20 | Q | 2 | 0.728 | 0.164 | 0.134 | 0.375 | 1595.412 | 31.819 |
|  | 21 | Q | 2.5 | 0.727 | 0.159 | 0.141 | 0.397 | 1596.608 | 33.015 |
|  | 22 | Q | 3 | 0.724 | 0.159 | 0.172 | 0.379 | 1597.976 | 34.383 |
|  | 23 | Q | 3.5 | 0.725 | 0.158 | 0.188 | 0.373 | 1599.556 | 35.963 |
|  | 24 | Q | 4 | 0.722 | 0.160 | 0.188 | 0.373 | 1601.314 | 37.721 |
|  | 23 | Q | 3.5 | 0.879 | 0.265 | 0.069 | 0.333 | 499.931 | 2.084 |
|  | 24 | Q | 4 | 0.812 | 0.222 | 0.069 | 0.333 | 500.569 | 2.723 |

Continuation of **Table S3**

| Modelling instance | Model | fc | rm | avg AUC | sd AUC | avg OR 10 pct | sd OR 10 pct | AICc | delta AICc |
| --- | --- | --- | --- | --- | --- | --- | --- | --- | --- |
| CS-Ch-Ar | 1 | L | 0.5 | 0.803 | 0.118 | 0.108 | 0.188 | 7352.438 | 159.208 |
|  | 2 | L | 1 | 0.811 | 0.119 | 0.119 | 0.210 | 7359.033 | 165.803 |
|  | 3 | L | 1.5 | 0.812 | 0.124 | 0.108 | 0.188 | 7367.472 | 174.242 |
|  | 4 | L | 2 | 0.817 | 0.123 | 0.104 | 0.180 | 7366.903 | 173.673 |
|  | 5 | L | 2.5 | 0.819 | 0.121 | 0.108 | 0.188 | 7367.884 | 174.654 |
|  | 6 | L | 3 | 0.823 | 0.121 | 0.104 | 0.180 | 7369.023 | 175.793 |
|  | 7 | L | 3.5 | 0.822 | 0.127 | 0.108 | 0.173 | 7368.150 | 174.920 |
|  | 8 | L | 4 | 0.821 | 0.121 | 0.104 | 0.180 | 7369.266 | 176.035 |
|  | 9 | Q | 0.5 | 0.797 | 0.110 | 0.127 | 0.224 | 7341.634 | 148.404 |
|  | 10 | Q | 1 | 0.807 | 0.114 | 0.131 | 0.232 | 7356.795 | 163.564 |
|  | 11 | Q | 1.5 | 0.801 | 0.114 | 0.127 | 0.224 | 7356.085 | 162.855 |
|  | **12** | **Q** | **2** | **0.858** | **0.093** | **0.101** | **0.207** | **7193.230** | **0.000** |
|  | 13 | Q | 2.5 | 0.806 | 0.112 | 0.127 | 0.224 | 7358.180 | 164.950 |
|  | 14 | Q | 3 | 0.806 | 0.112 | 0.123 | 0.226 | 7358.363 | 165.133 |
|  | 15 | Q | 3.5 | 0.807 | 0.114 | 0.123 | 0.226 | 7360.908 | 167.678 |
|  | 16 | Q | 4 | 0.808 | 0.114 | 0.116 | 0.212 | 7363.731 | 170.501 |
|  | 17 | H | 0.5 | 0.838 | 0.081 | 0.157 | 0.208 | 7451.318 | 258.088 |
|  | 18 | H | 1 | 0.856 | 0.084 | 0.127 | 0.205 | 7252.816 | 59.586 |
|  | 19 | H | 1.5 | 0.853 | 0.088 | 0.108 | 0.207 | 7221.176 | 27.946 |
|  | 20 | H | 2 | 0.805 | 0.112 | 0.127 | 0.224 | 7358.380 | 165.150 |
|  | 21 | H | 2.5 | 0.857 | 0.097 | 0.112 | 0.214 | 7201.429 | 8.199 |
|  | 22 | H | 3 | 0.857 | 0.101 | 0.112 | 0.214 | 7199.231 | 6.001 |
|  | 23 | H | 3.5 | 0.856 | 0.102 | 0.112 | 0.214 | 7215.442 | 22.212 |
|  | 24 | H | 4 | 0.854 | 0.104 | 0.112 | 0.214 | 7212.966 | 19.736 |
| TdF-SCh | 1 | L | 0.5 | 0.892 | 0.290 | 0.110 | 0.417 | 502.528 | 4.682 |
|  | 2 | L | 1 | 0.874 | 0.247 | 0.110 | 0.417 | 504.367 | 6.521 |
|  | 3 | L | 1.5 | 0.877 | 0.243 | 0.110 | 0.417 | 499.197 | 1.351 |
|  | 4 | L | 2 | 0.887 | 0.245 | 0.110 | 0.417 | 500.582 | 2.736 |
|  | 5 | L | 2.5 | 0.898 | 0.269 | 0.110 | 0.417 | 502.343 | 4.497 |
|  | 6 | L | 3 | 0.853 | 0.190 | 0.110 | 0.417 | 498.243 | 0.397 |
|  | 7 | L | 3.5 | 0.843 | 0.171 | 0.110 | 0.417 | 499.007 | 1.161 |
|  | 8 | L | 4 | 0.843 | 0.171 | 0.110 | 0.417 | 499.822 | 1.976 |
|  | 9 | LQ | 0.5 | 0.895 | 0.297 | 0.110 | 0.417 | 501.803 | 3.957 |
|  | 10 | LQ | 1 | 0.873 | 0.238 | 0.110 | 0.417 | 499.390 | 1.544 |
|  | **11** | **LQ** | **1.5** | **0.899** | **0.229** | **0.110** | **0.417** | **497.846** | **0.000** |
|  | 12 | LQ | 2 | 0.879 | 0.236 | 0.110 | 0.417 | 498.774 | 0.928 |
|  | 13 | LQ | 2.5 | 0.884 | 0.240 | 0.069 | 0.333 | 499.828 | 1.982 |
|  | 14 | LQ | 3 | 0.888 | 0.251 | 0.027 | 0.250 | 501.097 | 3.251 |
|  | 15 | LQ | 3.5 | 0.899 | 0.267 | 0.027 | 0.250 | 502.588 | 4.742 |
|  | 16 | LQ | 4 | 0.803 | 0.260 | 0.069 | 0.333 | 500.602 | 2.756 |
|  | 17 | Q | 0.5 | 0.893 | 0.260 | 0.069 | 0.333 | 511.373 | 13.527 |
|  | 18 | Q | 1 | 0.883 | 0.233 | 0.110 | 0.417 | 503.900 | 6.054 |
|  | 19 | Q | 1.5 | 0.878 | 0.227 | 0.110 | 0.417 | 498.365 | 0.519 |
|  | 20 | Q | 2 | 0.877 | 0.232 | 0.069 | 0.333 | 499.173 | 1.327 |
|  | 21 | Q | 2.5 | 0.876 | 0.233 | 0.027 | 0.250 | 500.172 | 2.326 |
|  | 22 | Q | 3 | 0.887 | 0.246 | 0.069 | 0.333 | 501.363 | 3.517 |
|  | 23 | Q | 3.5 | 0.879 | 0.265 | 0.069 | 0.333 | 499.931 | 2.084 |
|  | 24 | Q | 4 | 0.812 | 0.222 | 0.069 | 0.333 | 500.569 | 2.723 |

**Figure S1.** Phylogeographic structure of *Abrothrix olivacea* as inferred from their mitochondrial variation (see Quiroga-Carmona et al. 2022). A) Bayesian tree showing the genealogical relationships of the 122 *Cytb* haplotype classes. Bayesian posterior probability (PP) of each mitochondrial phylogroup and the major clades are provided. Terminal labels include the number of each haplotype class, the number (within parentheses) of specimens sharing the haplotype class, and the localities (within brackets) where each haplotype class occurs. Colored shadow at terminal labels identifies each phylogroup (green: N-Ch; yellow: CS-Ch-Ar; red: Men-Ar; blue: TdF-SCh). B) Map of southern South America depicting the collection localities (1-103) of the individuals included in this study. This plate was composed using QGIS, version 3.18.2-Zürich. Shape and color of the locality symbols differ among the main intraspecific mitochondrial phylogroups recognized (green diamond: N-Ch; yellow triangle: CS-Ch-Ar; red pentagon: Men-Ar; blue circle: TdF-SCh).


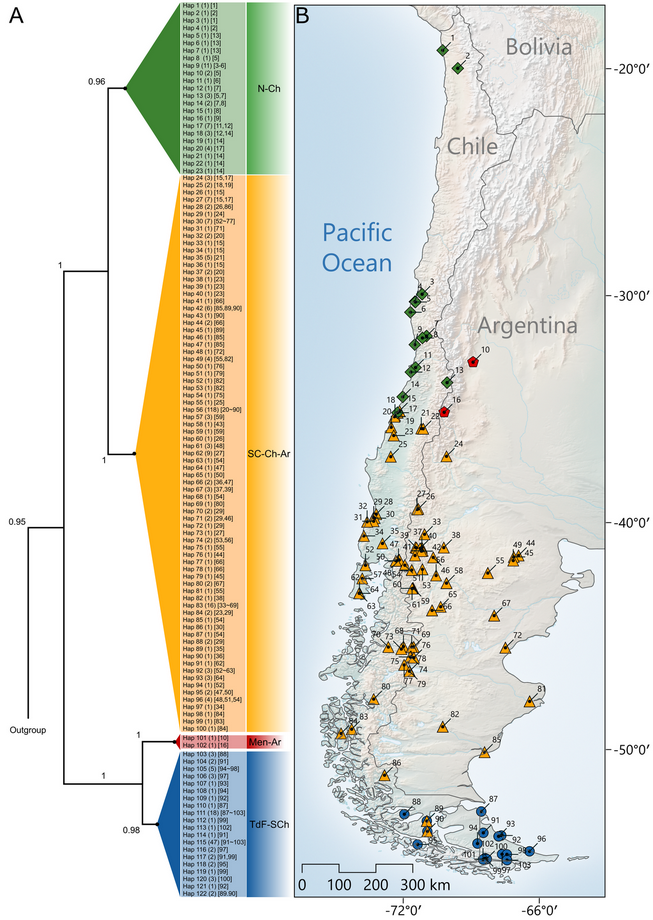


**Figure S2.** Bounded region of South America (extent: -18.00° N–63.50° W and -56.15° N–76.50° W) selected as the study region to train, calibrate and project the final ecological niche models. The spatially ﬁltered localities employed to modeling the ecological climatic niche of *Abrothrix olivacea* (A) and their mitochondrial phylogroups (B) are represented with circles (gray circles: *A*. *olivacea*; green circles: N-Ch; yellow circles: CS-Ch-Ar; blue circles: TdF-SCh). White symbols within locality circles in the panel A represent the localities where the sequenced individuals were captured (diamond: N-Ch; triangle: CS-Ch-Ar; circle: TdF-SCh). The areas delimited by each minimum convex polygon (blue thin lines) and its respective enveloping geographic buffers of 50 km (red dashed lines) are depicted for *A*. *olivacea* and their mitochondrial phylogroups. Maps were composed using QGIS, version 3.18.2-Zürich.


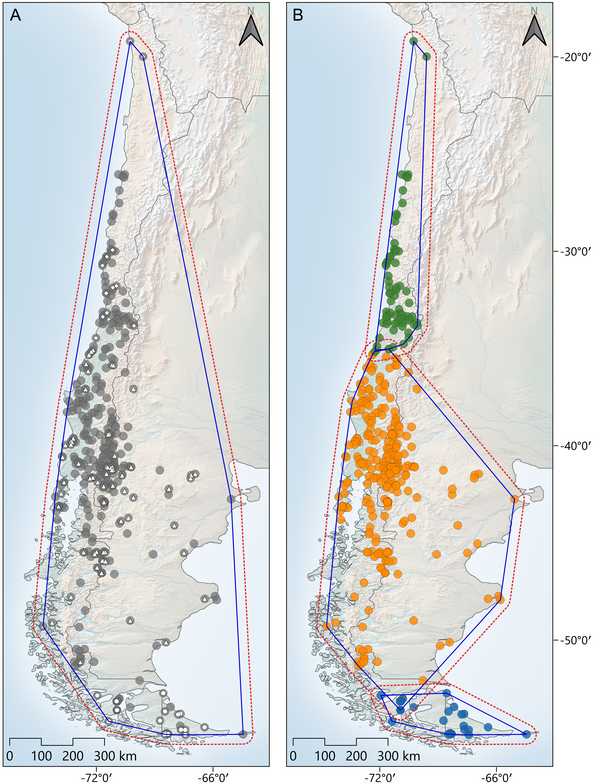


**Figure S3.** Genetic-based Principal Component Analysis (gPCA) performed with the 186 mt-SNPs obtained from 416 individuals of Abrothrix olivacea. Each frame (A-C) depicts the pair orthogonal projection of the three first principal components, which cumulate 69.79% of the explained variance. Individuals were grouped according to their belonging to each mitochondrial phylogroup, and these are colored as is depicted in the legend.

**
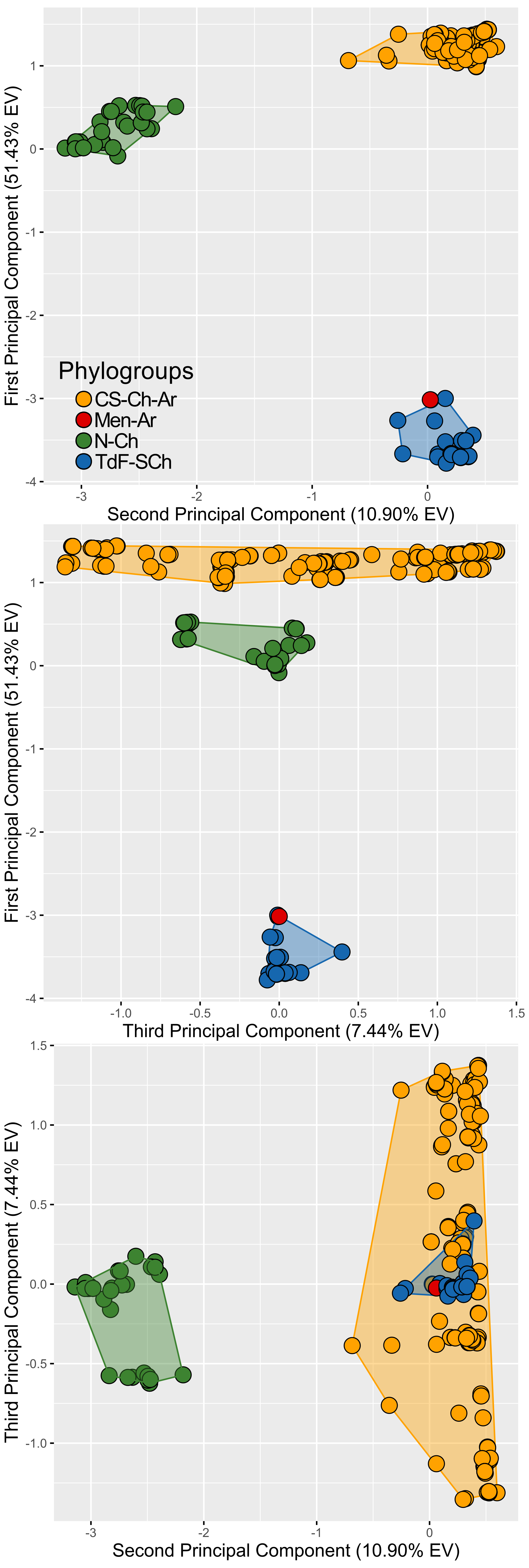
**

**Figure S4.** Mantel tests directed to evaluate isolation by distance model (IBD). Confidence interval, regression line, correlation coefficient (*R*) and their respective *p*-value are depicted for each implementation, corresponding to the entire dataset of *Abrothrix olivacea* (gray circles) and three of their mitochondrial phylogroups (green circles: N-Ch; yellow circles: CS-Ch-Ar; blue circles: TdF-SCh), as is indicated in the title of each panel.


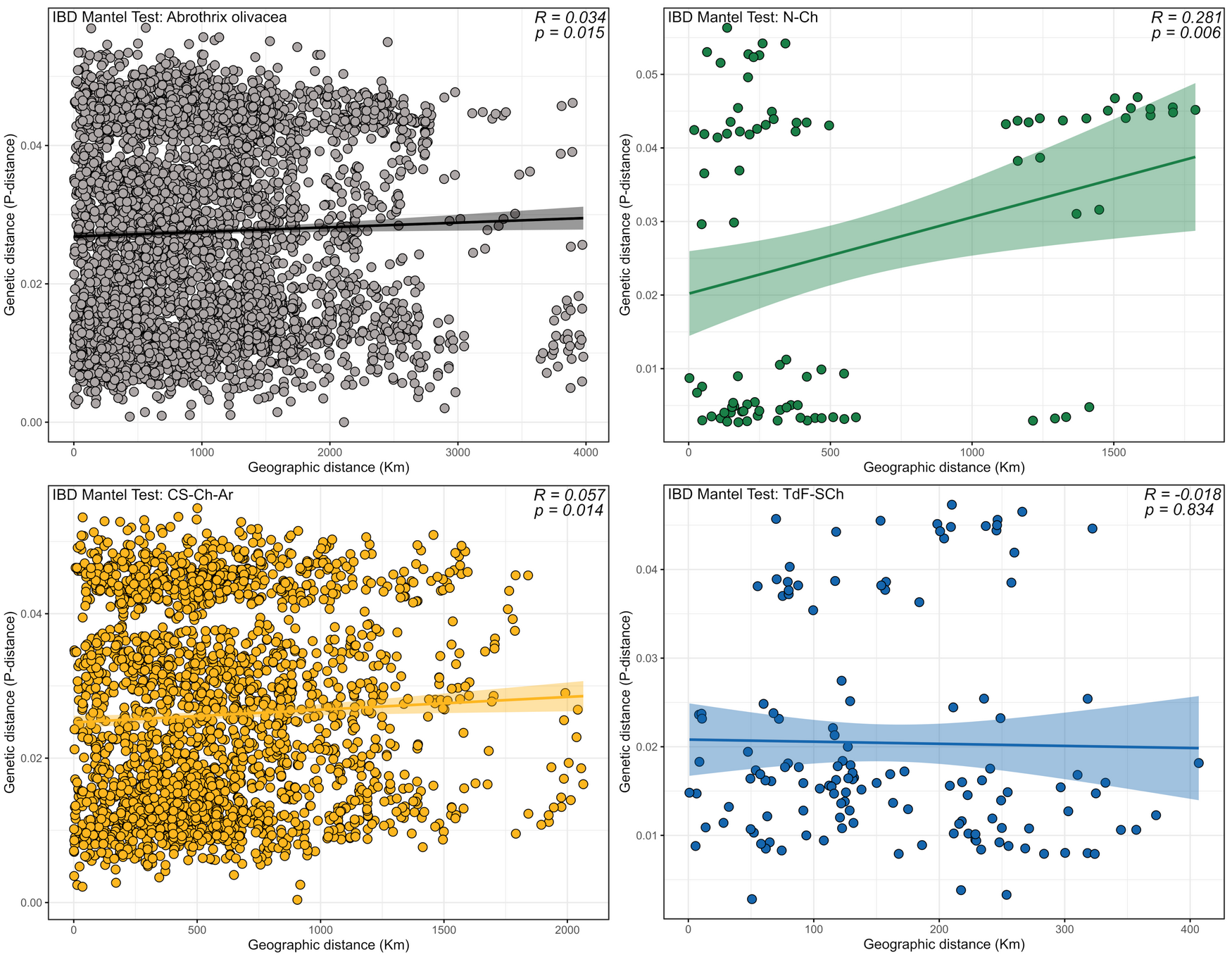


**Figure S5.** Mantel tests directed to evaluate isolation by environment model (IBE). Confidence interval, regression line, correlation coefficient (*R*) and their respective *p*-value are depicted for each implementation, corresponding to the entire dataset of *Abrothrix olivacea* (gray circles) and three of their mitochondrial phylogroups (green circles: N-Ch; yellow circles: CS-Ch-Ar; blue circles: TdF-SCh), as is indicated in the title of each panel.


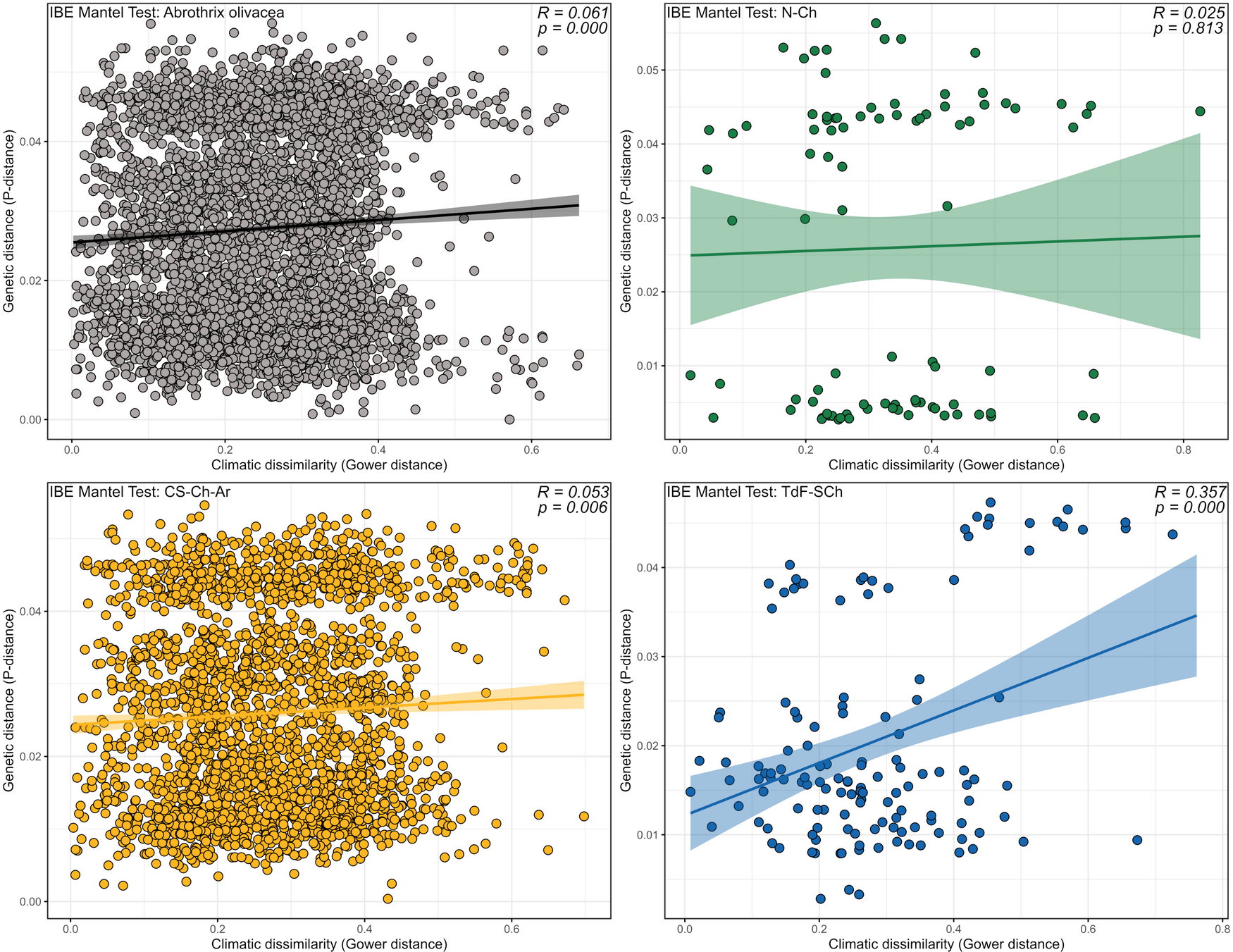


**Figure S6.** Results associated to the Procrustes analysis. The upper panel show a histogram of the Procrustes similarity scores (*t*), the value of the Procrustes similarity score (*t_0_*) and their statistical significance tested from the 10000 randomized permutations computing the similarities scores (*t*) between the gPCA coordinates and the geographic locations. The blue-dashed line indicates the *t_0_* value in the x axis. The lower panel shows a barplot built with the magnitude of each residual, which in this case can be considered as a proxy of deviation from the expected pattern of genetic variation based on geography. Thus, this implies that geographically more distant individuals are in turn genetically more different. The 416 residual bars are colored according to colors gave to the mitochondrial phylogroups in previous plots. The table inserted in the plot shows the results of the Post-Hoc Tukey Test for the ANOVA completed based on the magnitude of the residuals. Values of p ≤ 0.05 indicates that differences between means of residuals magnitude of each mitochondrial phylogroup are significant.


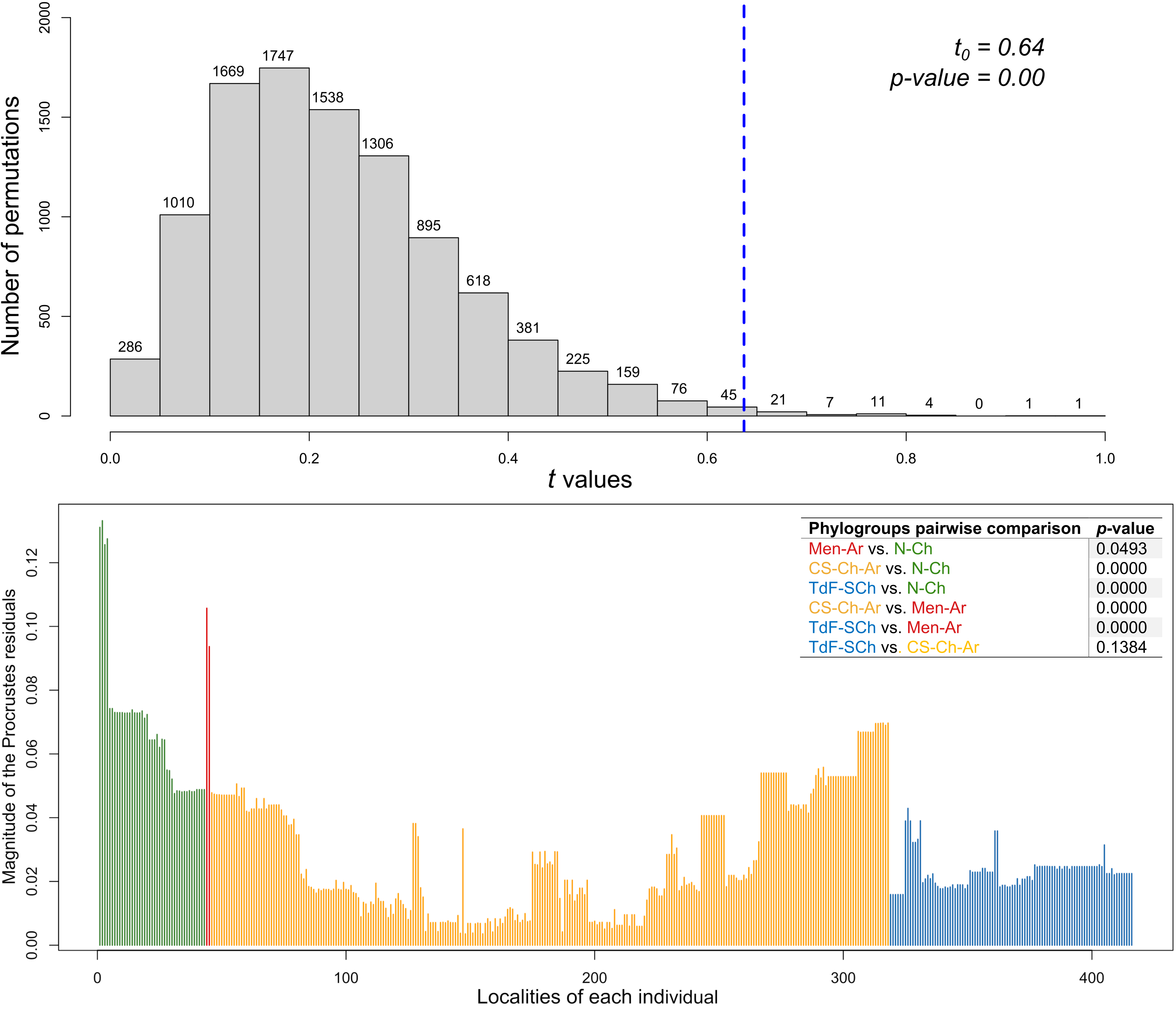


**Figure S7.** Principal Component Analysis (PCA) based on the characterization of the climatic niche of each mitochondrial phylogroup of Abrothrix olivacea. Each frame (A-C) depicts the pair of orthogonal projection of the three first principal components, which cumulate 88.97% of the explained variance (see Table 4). Localities were grouped according to the geographic distribution of each mitochondrial phylogroup, and groups are colored as is depicted in the legend.

**
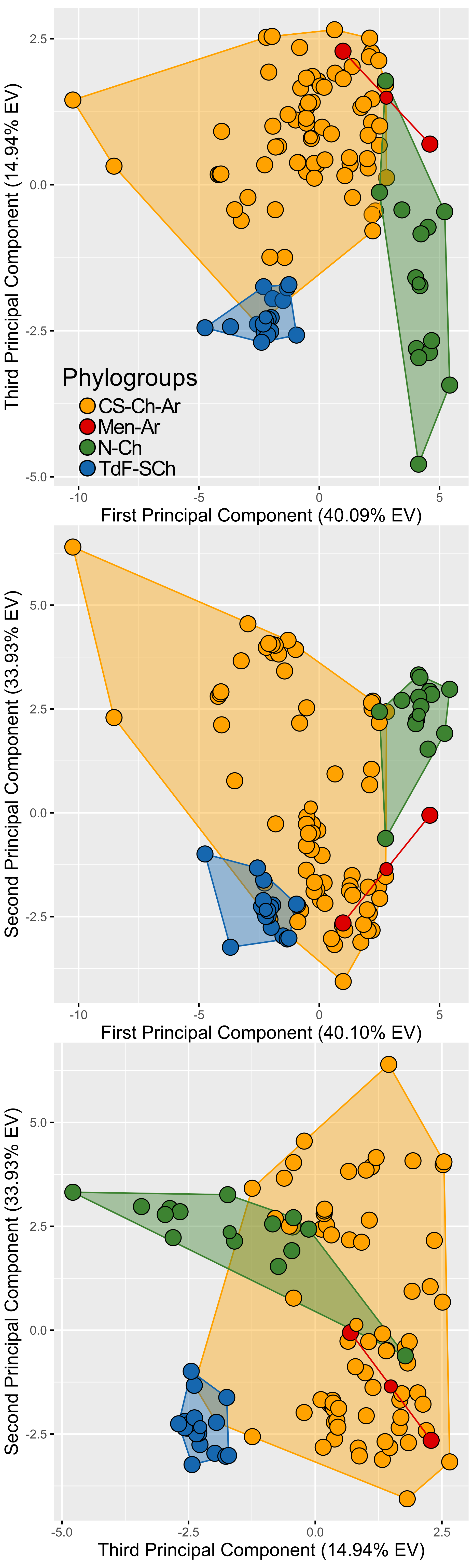
**

**Figure S8.** Spatial dynamics of the climatically suitable areas estimated for *Abrothrix olivacea* with the projection of the ecological niche models onto the climatic scenarios explored. Values of climatic suitability were discretized into three categories that summarize the upper component of the suitability interval (i.e., 0.25-1). Climatic scenarios explored correspond to conditions available during the Last Interglacial (LIG: ∼120-140 Kay BP), the Last Glacial Maximum (LGM: ∼22 Kay BP), the Mid Holocene (Mid-Holocene: ∼6-8 Kay BP), and the Current conditions (1960-1990). Quantification of area dynamics are presented in square kilometers (km^2^) and a graphical representation of this is depicted in the table inserted.


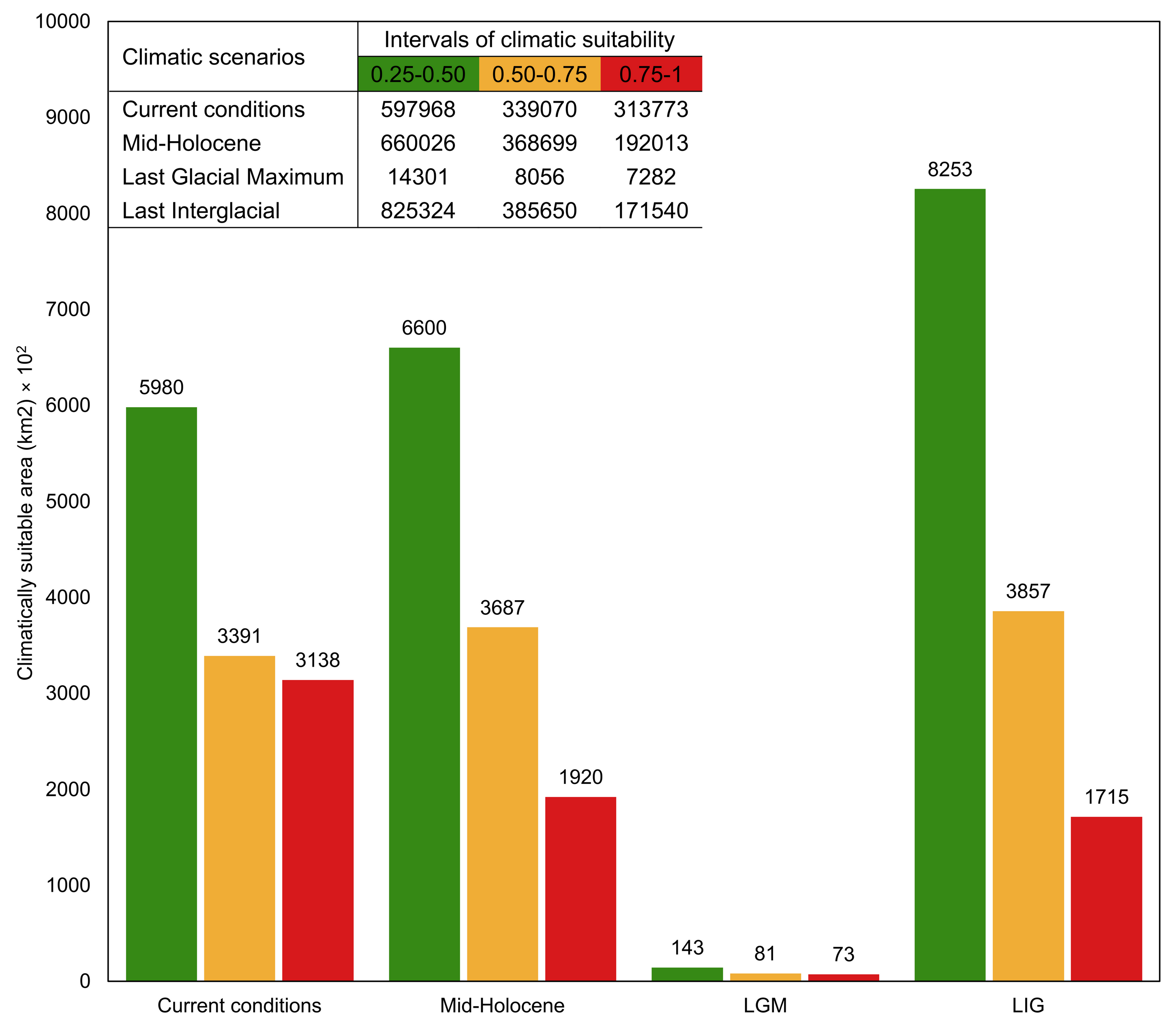


**Figure S9.** Potential geographic distribution of three (N-Ch, CS-Ch-Ar and TdF-SCh) of the mitochondrial phylogroups identified within *Abrothrix olivacea* in the climatic scenarios explored from present to past (Current conditions: 1960-1990; Mid Holocene: ∼6-8 Kay BP; Last Glacial Maximum: ∼22 Kay BP; Last Interglacial: ∼120-140 Kay BP). Each panel depicts the spatial projection of the climatic niche model constructed for each phylogroup in the climatic scenarios explored. The light blue shading represents the extent of the Patagonian Ice Sheet during the LGM according to McCulloch et al. (2000). Climatic-Environmental suitability values are depicted as continuous representation and areas with warmer colors indicate regions whit higher climatic suitability in each case. Maps were composed using QGIS, version 3.18.2-Zürich.


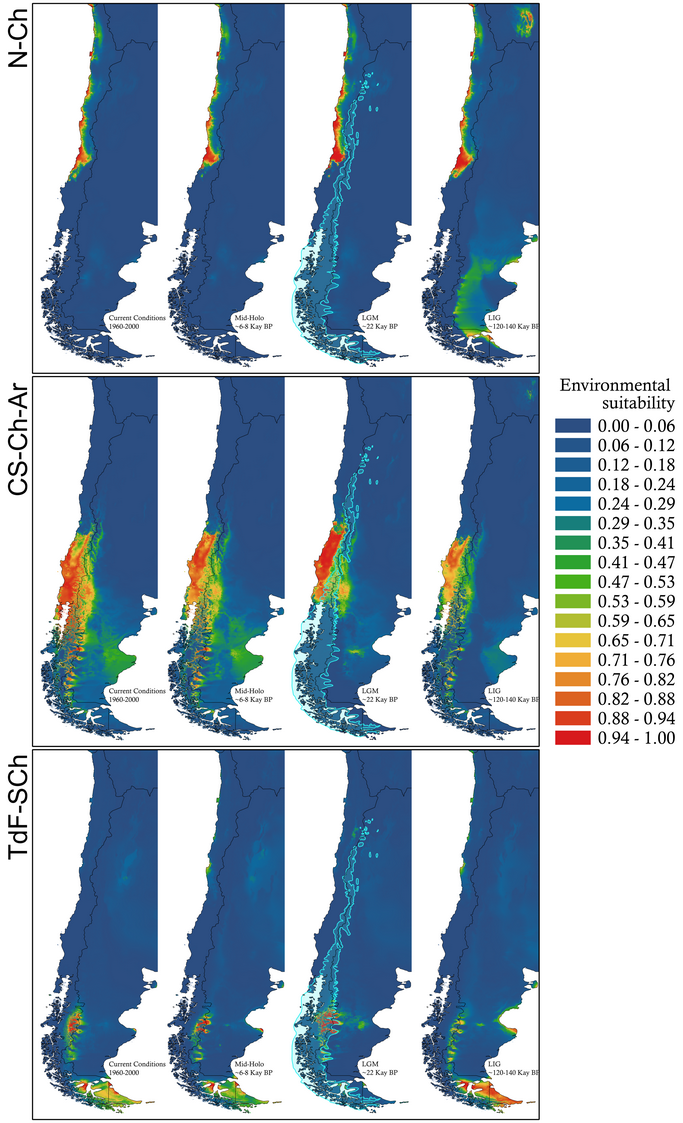


**Figure S10.** Geographic representation of the MESS analyses conducted to evaluate the uncertainty associated with the process of model transference. Results are depicted for the niche model transference conducted for *Abrothrix olivacea* and three of its mitochondrial phylogroups (N-Ch, CS-Ch-Ar, and TdF-SCh; see Figure S2 and S9). Similarity surfaces were estimated for each paleoclimatic scenario explored (Mid-Holo, LGM, and LIG), respect to climatic training conditions (i.e., Current climatic conditions). Analogue climatic conditions similar to native range (positive values) are represented from blue tones. Increasingly non-analogue climatic conditions (negative values) are indicated by red tones.


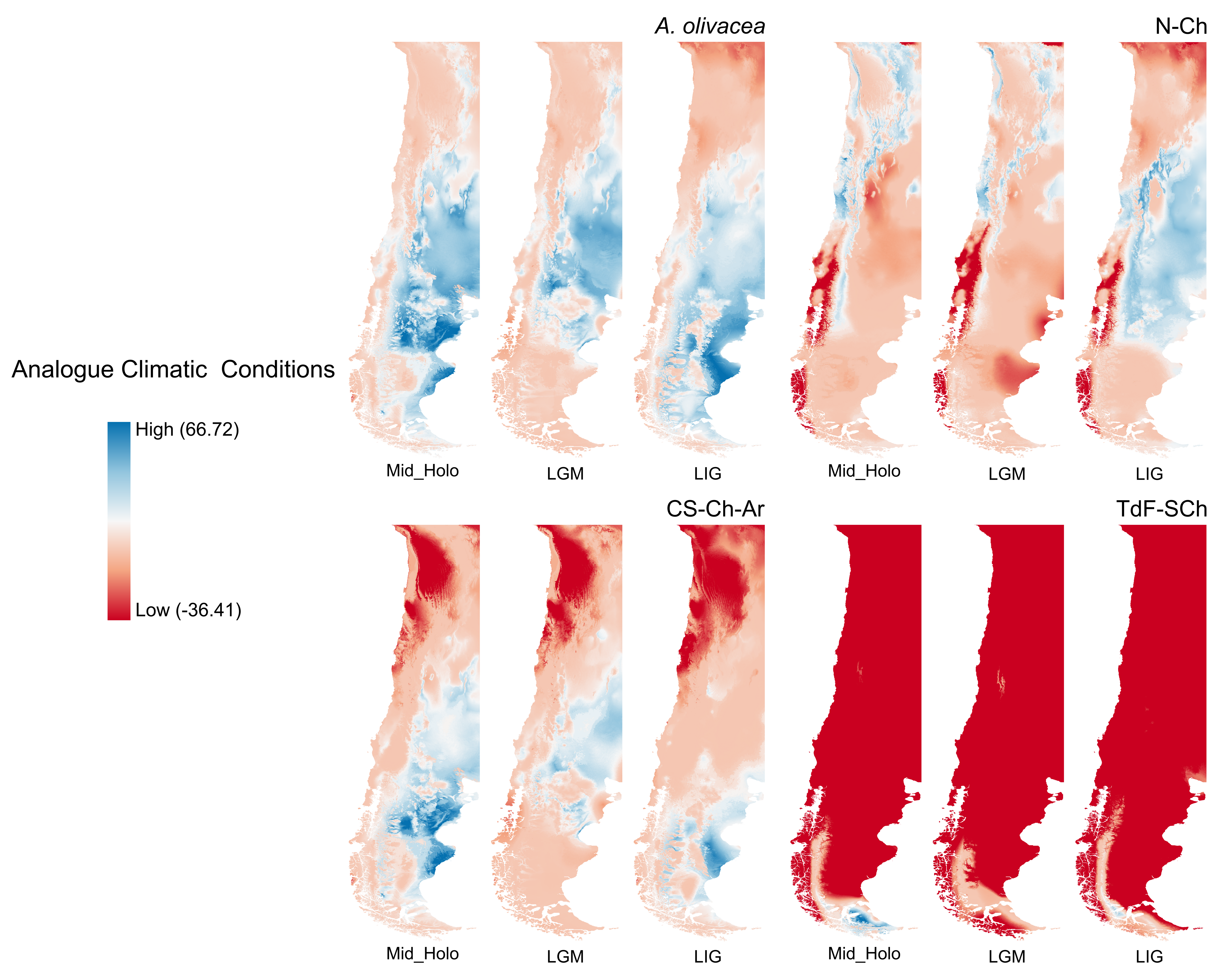

Supplement: Supplementary file 1 — Supplementary Information. [file 41598_2022_26937_MOESM1_ESM.docx]
